# A Targeted Metabolomic Assessment of Oral Glutathione Bioavailability and Safety in Humans: A Randomized Crossover Clinical Trial

**DOI:** 10.3390/antiox15030354

**Published:** 2026-03-11

**Authors:** Julia Solnier, Min Du, Yiming Zhang, Yoon Seok Roh, Yun Chai Kuo, Afoke Ibi, Simon Wood, Mary Hardy, Roland J. Gahler, Chuck Chang

**Affiliations:** 1 ISURA, Clinical Research, Burnaby, BC V3N 4S9, Canada; mdu@isura.ca (M.D.); yzhang@isura.ca (Y.Z.); kroh@isura.ca (Y.S.R.); aibi@isura.ca (A.I.); cchang@isura.ca (C.C.); 2 School of Public Health, Faculty of Health Sciences, Curtin University, Perth, WA 6845, Australia; simon.wood@ubc.ca; 3 InovoBiologic Inc., Calgary, AB Y2N 4Y7, Canada; 4 Food, Nutrition and Health Program, University of British Columbia, Vancouver, BC V6T 1Z4, Canada; 5 Association of Integrative and Holistic Medicine, San Diego, CA 92037, USA; mary@maryhardy.com; 6 Factors Group R & D, Burnaby, BC V3N 4S9, Canada

**Keywords:** glutathione, oral bioavailability, metabolites, safety, dietary supplements, nutraceuticals, pharmacokinetics, human study

## Abstract

Glutathione (GSH), often referred to as the “master antioxidant,” plays a vital role in protecting cells against oxidative stress. This human pilot study aimed to evaluate the oral absorption and safety profile of a novel formulation of micellar glutathione (LipoMicel^®^, LMG) compared with two commonly used dietary supplement forms: standard glutathione (STD) and liposomal glutathione (Setria^®^ Glutathione, LSG). In the first phase, a randomized, double-blind, crossover study was conducted in healthy adults (*n* = 14) to assess whole-blood GSH following single oral doses using baseline-adjusted pharmacokinetic parameters (incremental AUC_0–24_ [iAUC_0–24_], C_max_, T_max_) and a targeted panel of glutathione-related metabolites. In the second phase, a 30-day, single-arm follow-up assessed the safety and tolerability of the most bioavailable formulation (LMG) in the same participants. Compared with STD (500 mg), LMG (300 mg) produced significantly higher baseline-adjusted systemic GSH exposure and peak response (iAUC_0–24_: 1287.5 ± 179.0 vs. 517.8 ± 180.0 µg·mL·h; *p* = 0.0064; ΔC_max_: 103.9 ± 11.8 vs. 42.8 ± 11.5 µg/mL; *p* = 0.0003), corresponding to ~2.49-fold higher incremental exposure and ~2.43-fold higher peak response at the administered doses. When dose-normalized to a 300 mg equivalent, the incremental exposure (iAUC) and C_max_ were up to 4-fold higher for LMG than STD. In the targeted metabolite panel, most analytes showed no formulation-dependent differences; however, dose-normalized methionine exposure was significantly higher with LMG than STD (iAUC: 149.9 ± 30.8 vs. 32.7 ± 28.3 µg·mL·h; *p* = 0.0151; ~4.58-fold). No significant differences were observed in oxidized glutathione (GSSG) exposure, while the GSH/GSSG ratio was higher following LMG versus STD (*p* = 0.001). No significant changes in clinical safety markers (e.g., ALT, AST, ALP, creatinine) were observed following 30 days of daily LMG administration at 600 mg/d. The novel micellar glutathione formulation demonstrated enhanced oral bioavailability compared with a standard glutathione preparation and was well tolerated over 30 days in healthy adults. These findings present LipoMicel^®^ as a promising approach for oral glutathione delivery and warrant further investigation into its long-term physiological and clinical effects. This clinical trial was registered at ClinicalTrials.gov under trial ID NCT06345950 on 3 April 2024.

## 1. Introduction

**Figure 1:**
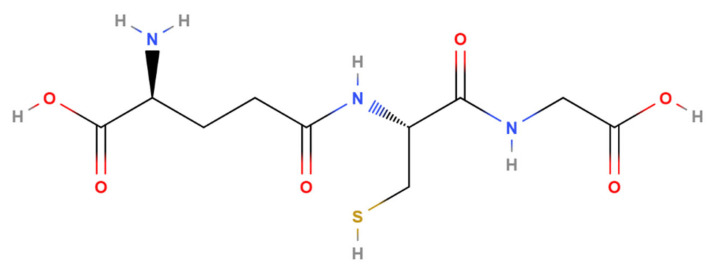
Chemical structure of glutathione (C10H17N3O6S; molecular weight: 307.33; source: PubChem). Atoms colors are as follows: carbon atoms are shown in black, oxygen in red, nitrogen in blue, hydrogen in grey and sulfur in yellow. The thiol (-SH) group on the cysteine side chain (yellow sulfur) is the key functional group responsible for glutathione’s biological activity.

Glutathione (GSH) is an important water-soluble endogenous antioxidant structured as a low-molecular-weight tripeptide containing a thiol group that is essential for its biological activity [1] (Figure 1). It is synthesized intracellularly from glutamic acid, cysteine, and glycine in mammalian tissues; in the human body, its synthesis is primarily carried out by the liver. GSH plays diverse physiological roles in cellular defense, detoxification, and various metabolic and signaling pathways [2,3]. For example, it plays a pivotal role in regenerating other antioxidants, such as tocopherols and ascorbate [4]. Additionally, it also functions as a thiol buffer for various cellular proteins such as metallothioneins and thioredoxins, and as a key cofactor for numerous enzymes [3].

Research shows that endogenous GSH levels naturally decrease with age and in association with chronic disease, contributing to decreased mitochondrial health, increased oxidative burden (cellular damage) and metabolic dysregulation [5]. Having garnered significant attention due to numerous positive health implications for the immune system, the cardiovascular system, and the respiratory system (e.g., GSH deficiency is associated with severe COVID-19 outcomes), glutathione is being actively explored as an important antioxidant health supplement [6,7,8,9,10,11,12,13,14,15,16].

While intravenous delivery of glutathione may be the most efficient route as it can bypass metabolic barriers, oral delivery remains the most practical and accessible route for long-term use due to higher patient compliance [9,17]. Thus, it is immensely worthwhile to explore and develop as a preventive strategy. For use as a health supplement, oral dosage forms of GSH have many advantages in that they are easier to take, are minimally invasive and are more readily available without the need for a medical prescription [18]. These ease-of-use advantages allow oral dosage forms of GSH to be readily available and well suited for preventive use and as an adjunct in treating various conditions. Although oral supplementation with GSH presents an efficient and convenient route of administration with high patient compliance, physiological barriers to effective use remain. For example, oral glutathione supplementation faces absorption challenges in the gastrointestinal environment such as poor stability and rapid degradation by the intestinal enzyme γ-glutamyl transpeptidase (GGT) [18,19]. Consequently, only modest increases in blood concentrations are typically detected post-oral administration.

Researchers have attempted to overcome these biochemical challenges using different strategies to orally deliver the GSH molecule in its active form. The most commonly studied approaches to make it more stable are modifying the molecular structure of glutathione into S-acetyl glutathione, S-allyl glutathione, and l-cysteine–glutathione mixed disulfide [16,18,20], or co-administering GSH with permeation enhancers (e.g., chitosan, citric acid, cyclodextrins, glycerides, lauryl carnitine chloride, and sodium lauryl sulfate [18]. Enzymatic inhibitors, and delivery carriers such as nanoparticles, liposomes, microemulsions, and niosomes have also been investigated [18,21,22]. Furthermore, N-acetyl cysteine (NAC) has been revealed to be effective for increasing GSH levels in the blood [23,24] since it is a precursor of cysteine which is used for glutathione synthesis in human red blood cells [25]. However, the effectiveness of NAC supplementation relies on the body’s capacity to produce glutathione from precursor molecules, a process limited by age and potentially genetic factors or health conditions such as liver dysfunction [23]. Together, these observations suggest that oral glutathione supplementation may influence and be influenced by sulfur amino acid metabolism more broadly, rather than by simply increasing circulating GSH concentrations.

Oral bioavailability is typically assessed using AUC, C_max_, and T_max_. Interpretation is more complex for glutathione, which is endogenously present and tightly regulated; baseline concentrations vary between individuals and across study periods, and post-dose profiles reflect both supplementation and background turnover. For this reason, baseline-adjusted (incremental) exposure metrics can improve comparability across treatments. Given glutathione’s rapid degradation and intercompartmental redistribution, changes in glutathione-related metabolites may offer additional evidence of absorption and systemic handling.

Mammalian systems maintain low extracellular glutathione (GSH) concentrations [26] through rapid enzymatic degradation by GGT, which cleaves GSH into cysteinyl glycine and glutamate. GSH is replenished intracellularly via de novo synthesis from γ-glutamyl cysteine and glycine or through reduction of glutathione disulfide (GSSG) [7]. Due to this tight homeostatic regulation and rapid extracellular turnover, circulating blood GSH levels alone may not fully capture oral bioavailability. Accordingly, assessment of glutathione absorption may be strengthened by a targeted evaluation of glutathione-related metabolites that reflect complementary biological domains, including redox balance, γ-glutamyl cycle activity, sulfur amino acid availability, and hepatic sulfur utilization. Numerous intermediates as well as co-existing metabolites are generated from glutathione metabolism in mammalian systems [8,27,28,29,30]. After absorption into the bloodstream, GSH may be promptly anabolized or catabolized into other metabolic forms, several of which can be present simultaneously and interconvert between one another in order to maintain homeostasis [8,26]. Therefore, the addition of exogenous, supplemented GSH may temporarily affect the balance of metabolites during the course of absorption. Two of the most common measurements are the concentrations of GSH (glutathione in its reduced form) and GSSG (glutathione disulfide, a metabolite of glutathione in its oxidized form), which co-exist at varying ratios to function as a redox buffer [8,29,31,32]. In fact, due to this tightly controlled redox buffer, GSH and GSSG are often studied in terms of their sum (as total glutathione) and/or their ratio to each other [8,26,29,31,33]. However, beyond reduced and oxidized glutathione, several metabolites provide relevant and interpretable information in this context. l-cystine reflects extracellular redox status and cysteine availability for intracellular glutathione synthesis [34,35,36]. l-pyroglutamic acid (5-oxoproline) is an intermediate of the γ-glutamyl cycle that can accumulate under conditions of impaired glutathione synthesis or recycling and has been associated with metabolic stress and safety considerations [37,38,39,40]. Glutamate serves both as a structural component of glutathione and as a product of its extracellular turnover [19]. Methionine links glutathione metabolism to transsulfuration and one-carbon metabolism, while taurocholate, a taurine-conjugated bile acid, reflects hepatic utilization of cysteine-derived sulfur and bile acid handling [41,42,43]. Collectively, these metabolites offer a targeted, systems-level perspective on glutathione bioavailability and metabolic impact.

Prior studies of oral glutathione have largely focused on glutathione concentrations or redox ratios, with relatively few incorporating broader glutathione-related metabolites into pharmacokinetic evaluations of bioavailability in humans. In this pilot study, we compared three oral formulations—a novel micellar glutathione (LMG), standard glutathione (STD), and liposomal glutathione (LSG)—using 24 h baseline-adjusted PK parameters and a targeted metabolite panel to contextualize systemic handling of glutathione. Administered doses differed across arms to evaluate whether the micellar formulation could deliver higher incremental exposure at a lower dose. Thus, both as-dosed and dose-normalized baseline-adjusted PK outcomes are presented. Safety and tolerability of LMG were further assessed in a 30-day single-arm extension.

## 2. Materials and Methods

The study consisted of two segments: a 24 h pharmacokinetic (PK) assessment and a subsequent 30-day safety and tolerability evaluation. Both phases were conducted from June 2022 to June 2023 at a certified research facility (ISURA, Burnaby, BC, Canada) under standardized conditions, with study oversight provided by qualified research personnel. Ethical approval was granted by the Institutional Review Board (IRB) of the Canadian SHIELD Ethics Review Board (OHRP Registration IORG0003491; FDA Registration IRB00004157; approval letter ID 2022-04-003; approved on 21 June 2022). The study protocol, along with related information, was registered at ClinicalTrials.gov (ID# NCT06345950) on 3 April 2024 and conducted per the ethical standards as outlined in the Helsinki Declaration of 1975.

### 2.1. Participants

Participants were required to meet the criteria below to be eligible for enrollment:

- Healthy male or female.
- Aged 21 years or older.

The exclusion criteria were as listed below:

- Pregnancy.
- The use of anti-inflammatory or non-steroidal anti-inflammatory drugs.
- Previous history of cardiovascular or liver disease or symptomatic chronic inflammatory disease.
- The use of antioxidant supplements or cholesterol-lowering agents.
- Change in diet habits or lifestyle of the participants (diet, physical activity, etc.) throughout the study period.
- Alcohol intake (>20 g/day).
- Use of nicotine or tobacco or cannabis.
- Participation in another investigational study.

An online health questionnaire regarding each participant’s medical history was completed by all participants upon study enrollment. Voluntary, written, and informed consent was mandatory to participate in the study.

### 2.2. Interventions

The following oral formulations of glutathione (reduced forms) were investigated regarding their pharmacokinetics:

- Micellar Glutathione (LipoMicel^®^; Natural Factors, Burnaby, BC, Canada): contains 300 mg l-glutathione per soft-gel capsule. Herein referred to as “LMG”.
- Liposomal Glutathione (Setria^®^; BioAbsorb Nutraceuticals, Richmond Hill, ON, Canada): contains 300 mg l-glutathione per soft-gel capsule. Herein referred to as “LSG”.
- Standard Glutathione (NOW Foods, Guelph, ON, Canada): contains 250 mg l-glutathione per hard-gel capsule, administered at a higher daily dose of 500 mg, i.e., 2 capsules. Herein referred to as “STD”.

All capsules were stored at room temperature, protected from heat, moisture, and direct light.

### 2.3. Outcomes

The primary outcomes were baseline-adjusted pharmacokinetic parameters for whole-blood glutathione over 24 h following a single dose: incremental area under the concentration–time curve from 0 to 24 h (iAUC_0–24_), baseline-adjusted maximum concentration (ΔC_max_), and time to maximum concentration (T_max_). For the targeted metabolite panel, corresponding 0–24 h exposure metrics (iAUC_0–24_, ΔC_max_, T_max_) were derived where applicable. Because administered doses were not identical across all arms, primary comparisons were performed using both as-dosed baseline-adjusted parameters and dose-normalized baseline-adjusted parameters (e.g., iAUC_0–24_ per mg and ΔC_max_ per mg).

Secondary outcomes included changes in clinical chemistry and electrolyte measures assessed pre-dose and at 24 h post-dose, and safety/tolerability outcomes during the 30-day extension (e.g., hepatic and renal function biomarkers).

### 2.4. Pharmacokinetic Study

**Figure 2:**
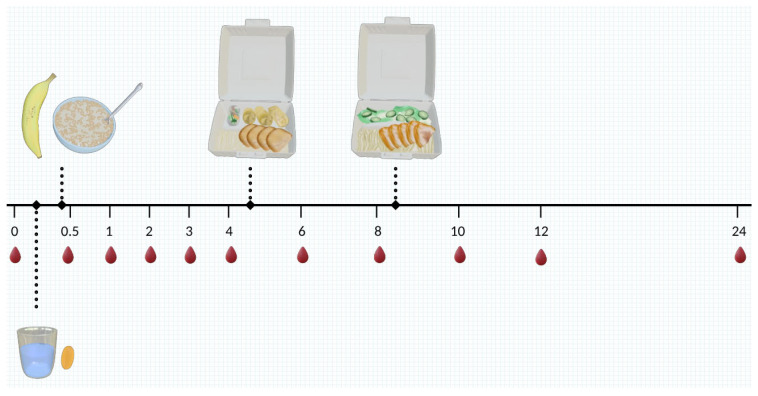
Treatment progress diagram for the pharmacokinetics portion of the study. Participants arrive after a 9 h fast. Intervention was given with water followed by breakfast. Lunches and dinners were provided at 4 h and 8 h after treatment. Blood was sampled from participants at 0, 0.5, 1, 2, 3, 4, 6, 8, 10, 12, and 24 h.

A randomized, double-blind, crossover study was conducted on healthy adults. Sample size was determined based on feasibility and consistency with prior exploratory pharmacokinetic studies [22,44]. A one-week washout period between treatments was implemented to minimize potential carry-over effects and was deemed sufficient based on in-house pre-trials. Participants received three different oral glutathione formulations across study visits, with administered doses determined a priori based on formulation type and commercially relevant supplement strengths: LipoMicel^®^ glutathione (LMG, 300 mg), liposomal glutathione (LSG, 300 mg), and standard unformulated glutathione (STD, 500 mg). Study treatments were administered in the morning with approximately 200 mL of water and a standardized breakfast under direct supervision by the study personnel. Prior to dosing (t = 0), baseline concentrations of glutathione and related metabolites were determined from fasting whole-blood samples. Capillary whole-blood samples were collected under supervision by fingertip lancet at pre-dose (baseline) and at 0.5, 1, 2, 3, 4, 6, 8, 10, 12, and 24 h post-dose. These samples were used for pharmacokinetic analysis of glutathione and a targeted panel of glutathione-related metabolites. Figure 2 illustrates the dosing schedule and blood sampling scheme for the pharmacokinetic phase of the study.

### 2.5. Safety and Tolerability

The biochemical safety profile of the newly developed micellar glutathione formulation (LMG), designed for enhanced bioavailability, was evaluated over a 30-day period in the same participants using a single-arm design. Given the pilot nature of the study, the sample size aligned with prior safety single-arm evaluations, to characterize common adverse events and evaluate within-participant changes in routine clinical safety biomarkers [45]. Routine blood chemistry parameters were monitored, including electrolytes, hepatic enzymes (e.g., AST, ALT, ALP), and renal markers (e.g., creatinine, GFR). The safety evaluation also included adverse events monitoring, as reported through structured health questionnaires.

### 2.6. Blood Collection and Processing

For pharmacokinetics, 50 µL of capillary whole blood was collected into pre-labeled capillary blood collection tubes coated with EDTA (Microvette^®^ POCT 50 µL K3E, Sarstedt, Germany). After the sample collection, whole blood was frozen immediately at –20 °C to keep it stable. All blood samples were processed and analyzed immediately.

For the 30-day safety assessment, venous blood samples were collected biweekly—at baseline, week 2, and week 4—by a certified phlebotomist from LifeLabs (Diagnostic Laboratory Mobile Services, Burnaby, BC, Canada) at the ISURA Research Facility (Burnaby, BC, Canada). Standard venipuncture procedures and validated clinical laboratory methods were used for the assessment of routine safety biomarkers, including hepatic, renal, hematological, and metabolic parameters.

### 2.7. Statistical Analysis

Pharmacokinetic comparisons of glutathione (GSH), total glutathione, related metabolites, and the reduced-to-oxidized glutathione (GSH/GSSG) ratio among the tested formulations were conducted in this crossover study using subject-level PK parameters. All randomized participants were included in the primary analyses according to the treatment received in each study period. Raw data were baseline-corrected to calculate incremental concentration parameters, followed by dose-correction of the STD group for comparison. Treatment effects were assessed using the ANOVA linear mixed-effects model (treatment as a fixed effect and subject as a random effect). For post hoc testing, Tukey’s multiple comparisons test was applied. Prior to analysis, the Shapiro–Wilk test was used to assess normality for all dependent variables; non-normally distributed data were log-transformed as appropriate. Safety-related blood chemistry and electrolyte data were analyzed using repeated-measures ANOVA. All data are presented as means ± standard error of the mean (SEM), and a *p*-value ≤ 0.05 was considered statistically significant. Statistical analyses and figure generation were conducted using GraphPad Prism, version 10.6 (GraphPad Software, San Diego, CA, USA).

#### Randomization and Blinding

Participants were randomized to one of the prespecified treatment sequences for this three-period crossover trial using a computer-generated randomization list using Microsoft Excel, without stratification or blocking. Briefly, treatments were coded as “A”, “B” and “C” and listed serially and column-wise for each participant. Three random numbers were then generated by the “=RAND()” function and pasted only as “Values” to preserve the number, next to the treatment codes. The codes were then sorted by the randomized numbers, from smallest to largest, to generate the allocation sequence for each study participant. An independent study assistant generated the allocation sequence and prepared participant-labeled dosing kits. Because the formulations differed in capsule type, participant blinding could not be fully guaranteed (participants were informed that they were taking different formulations of glutathione, but they were unaware of which specific capsule form corresponded to each formulation); however, allocation was concealed from the principal investigator and study personnel responsible for outcome assessment and data analysis until completion of analyses.

### 2.8. Determination of Glutathione (GSH) and Related Metabolites

Concentrations of whole-blood glutathione were determined as published previously [46] and revalidated on a Q-Exactive HRMS coupled to a Vanquish UHPLC (Thermo Fisher Scientific, Toronto, ON, Canada). Frozen blood samples were allowed to equilibrate at room temperature immediate to processing. Next, l-glutamic acid-2,3,3,4,4-d5 (98% assay, MS, Canada) was freshly dissolved in water at 3 µg/mL as an internal standard and added into each sample. Samples were then centrifuged at 16,000× *g* for 5 min at room temperature and then transferred onto a microplate for injection onto the analytical instrument.

UHPLC was carried out with a binary gradient consisting of 0.5% formic acid in water (A), and 0.5% formic acid in acetonitrile (B) using Acme Xceed C18, 100 mm × 2.1 mm, 1.9 µm columns (Phase Analytical Technology, State College, PA, USA) at a flow rate of 400 µL/min. LC-MS grade solvents and formic acid were obtained from Fisher Scientific (Durham, NC, USA). The gradient consists of an initial isocratic step of 98% A and 2% B for 4 min, which then progresses from 2% A to 65% B over 1.5 min. After that, the column is rinsed with 100% B for 1 min and then equilibrated at 2% B for 4.0 min before the next injection. The Orbitrap mass spectrometer was calibrated according to vendor specifications with a typical mass deviation over the course of a week of less than 2 ppm.

Data were collected in Full MS mode at a resolution setting of 70,000 and scanning 110–1000 *m*/*z* with a heated ESI source set to instrument defaults for a 400 µL/min flow rate. ThermoFisher Xcalibur 4.3 and TraceFinder 5.1 software were used to quantify protonated [M+H]^+^ adducts using a mass tolerance window of 5.00 ppm. The Glutathione Reference Standard (Millipore Sigma, Oakville, ON, Canada) was used as the chemical standard using a 6-point calibration curve (R^2^ > 0.996), and the concentrations of glutathione metabolites were determined relative to glutathione based on internal standard calibration.

## 3. Results

### 3.1. Baseline Data

**Figure 3:**
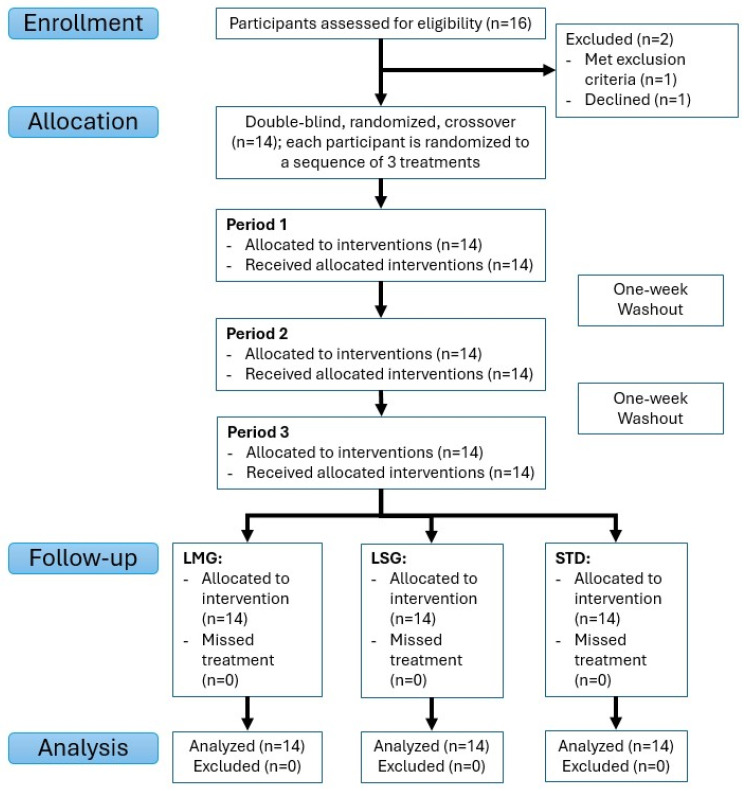
Participant flow diagram.

Participants were recruited during March 2022, with follow-up completed by June 2023. Table 1 describes the study population demographic data of participants who were assigned to treatment (*n* = 14). All participants received each assigned intervention as intended with no missed doses or protocol deviations (Figure 3).

### 3.2. Pharmacokinetics of the Different GSH Treatments

**Figure 4:**
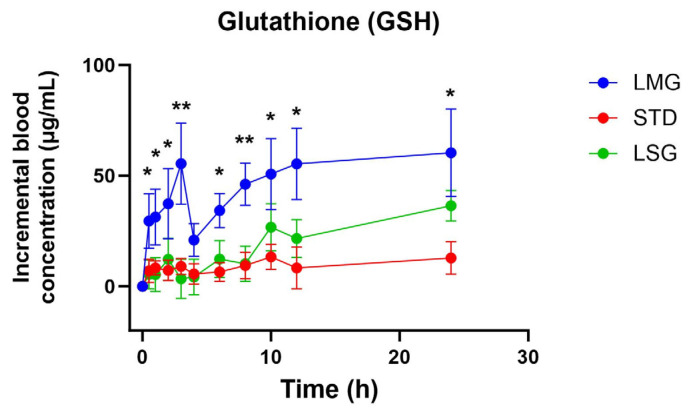
Blood concentration trend of glutathione (GSH) over 24 h. Data presented as mean ± SEM, *n* = 14. Data were analyzed using mixed-effects ANOVA with Tukey’s multiple comparisons test. Dose-corrected analysis of LMG (LipoMicel^®^) and LSG (Liposomal) administered at a single dose of 300 mg GSH vs. STD (standard) administered at a higher dose of 500 mg GSH; dose-normalized (300 mg equivalent). Significant differences between LMG and STD with *p* ≤ 0.05 are marked with a single asterisk (*) whereas differences with *p* ≤ 0.01 are marked with a double asterisk (**).

**Figure 5:**
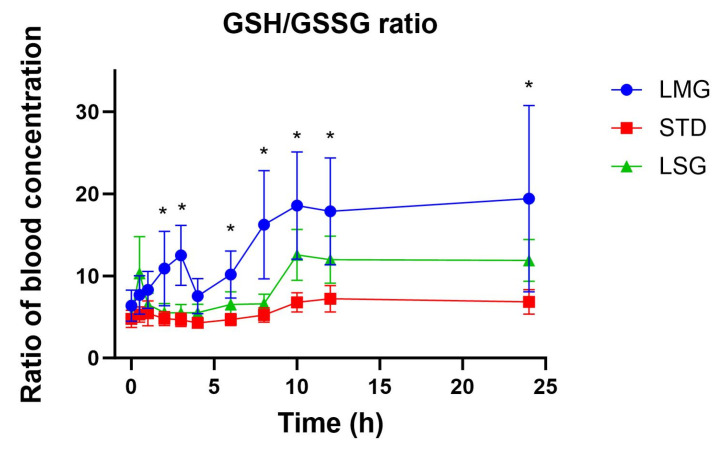
The ratio of GSH to GSSG in blood concentration is demonstrated. LMG shows a significant difference in GSH/GSSG ratio compared to STD (*p* = 0.001), but no significant difference when compared to LSG (*p* = 0.1478) using mixed-effects ANOVA with Tukey’s multiple comparisons test. Asterisks (*) denote statistically significant differences (*p* ≤ 0.05).

Pharmacokinetic parameters for reduced glutathione (GSH) are summarized in Table 2 and Table 3 and have been visualized as graphs as seen in Figure 4 and Figure 5. Significant treatment effects were observed for baseline-adjusted exposure and peak response (iAUC and ΔC_max_), while T_max_ did not differ between formulations. At the administered doses (LMG 300 mg; LSG 300 mg; STD 500 mg), LMG produced significantly higher iAUC and ΔC_max_ than STD (*p* = 0.0064 and *p* = 0.0003, respectively), corresponding to approximately 2.49-fold higher incremental exposure and 2.43-fold higher peak response (Table 3). To account for unequal dosing, parameters were dose-normalized and expressed as 300 mg equivalent values; under this normalization, LMG remained significantly higher than STD for both iAUC and ΔC_max_ (*p* = 0.0040 and *p* = 0.0003), corresponding to approximately up to 4-fold higher exposure and peak response at the 300 mg equivalent (Table 2). Per mg dose-normalized results are provided in Supplementary Materials Tables S1 and S2.

**Figure 6:**
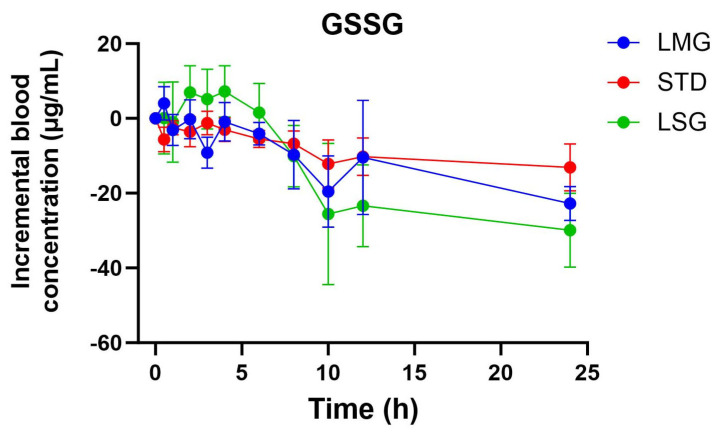
Blood concentration trend of GSSG over 24 h. Data presented as mean ± SEM, *n* = 14. The iAUC and ΔC_max_ did not differ significantly using mixed-effects ANOVA with Tukey’s multiple comparisons test; LMG (LipoMicel^®^) and LSG (Liposomal) administered at a single dose of 300 mg GSH vs. STD (standard) at a higher dose of 500 mg GSH. Data have been normalized for dose.

No significant formulation-dependent differences were observed for GSSG iAUC, ΔC_max_, or T_max_. However, the GSH/GSSG ratio was significantly higher following LMG compared with STD (Figure 5, *p* = 0.001), consistent with increased circulating reduced glutathione without a corresponding rise in GSSG (Figure 6, Table 4). Non-dose-corrected tables are presented in Supplementary Materials S3.

### 3.3. Targeted Metabolite Pharmacokinetics

**Figure 7:**
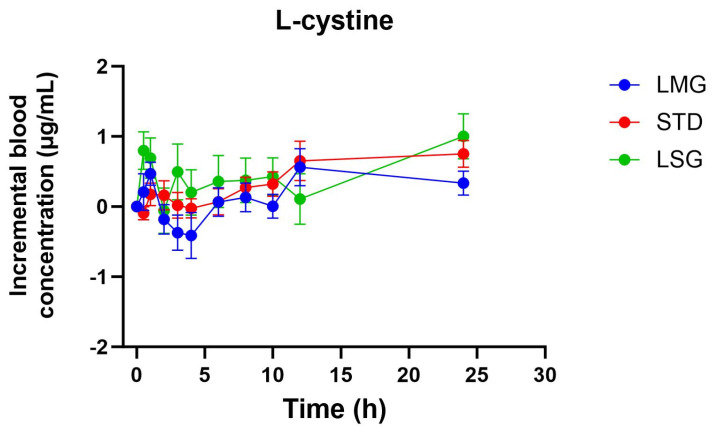
Blood concentration of l-cystine over 24 h. Data presented as mean ± SEM, *n* = 14. The iAUC and ΔC_max_ of LMG did not show significant differences compared to LSGs or STD using mixed-effects ANOVA with Tukey’s multiple comparisons test. LMG (LipoMicel^®^) and LSG (Liposomal) administered at a single dose of 300 mg GSH vs. STD (standard) at a higher dose of 500 mg GSH. Data have been normalized for dose.

**Figure 8:**
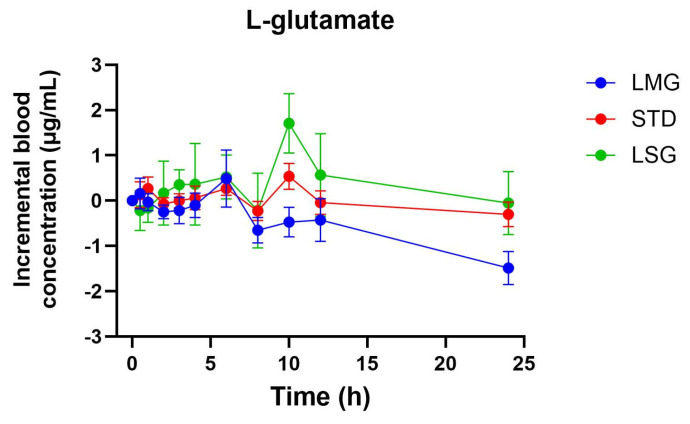
Blood concentration of l-glutamate over 24 h. Data presented as mean ± SEM, *n* = 14. The iAUC and ΔC_max_ of LMG did not show significant differences compared to LSGs or STD using mixed-effects ANOVA with Tukey’s multiple comparisons test. LMG (LipoMicel^®^) and LSG (Liposomal) administered at a single dose of 300 mg GSH vs. STD (standard) at a higher dose of 500 mg GSH. Data have been normalized for dose.

**Figure 9:**
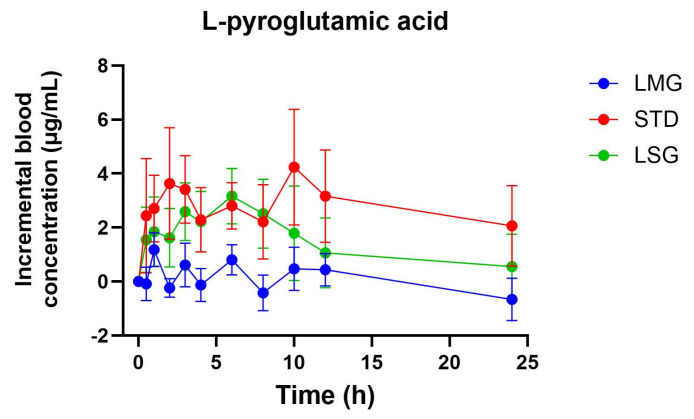
Blood concentration of l-pyroglutamic acid over 24 h. Data presented as mean ± SEM, *n* = 14. The iAUC and ΔC_max_ of LMG showed significant differences compared to LSGs or STD using mixed-effects ANOVA with Tukey’s multiple comparisons test. LMG (LipoMicel^®^) and LSG (Liposomal) administered at a single dose of 300 mg GSH vs. STD (standard) at a higher dose of 500 mg GSH. Data have been normalized for dose.

**Figure 10:**
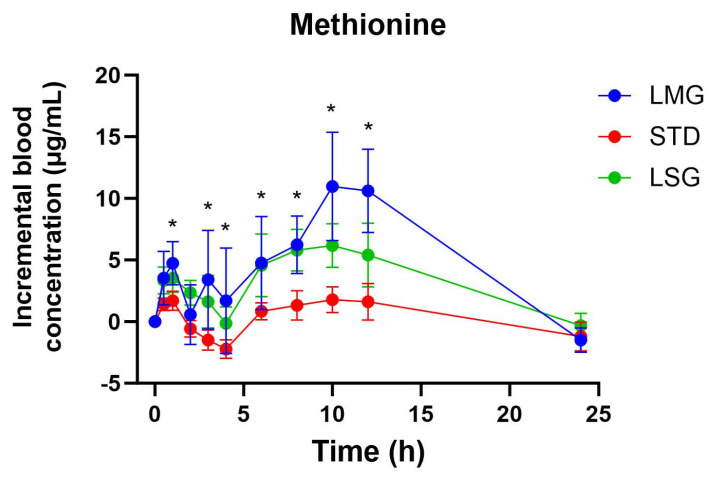
Blood concentration of methionine over 24 h. Data presented as mean ± SEM, *n* = 14. The iAUC or ΔC_max_ of LMG show significant differences compared to LSGs or STD using mixed-effects ANOVA with Tukey’s multiple comparisons test. LMG (LipoMicel^®^) and LSG (Liposomal) administered at a single dose of 300 mg GSH vs. STD (standard) at a higher dose of 500 mg GSH. Data have been normalized for dose. Asterisks (*) denote statistically significant differences (*p* ≤ 0.05).

**Figure 11:**
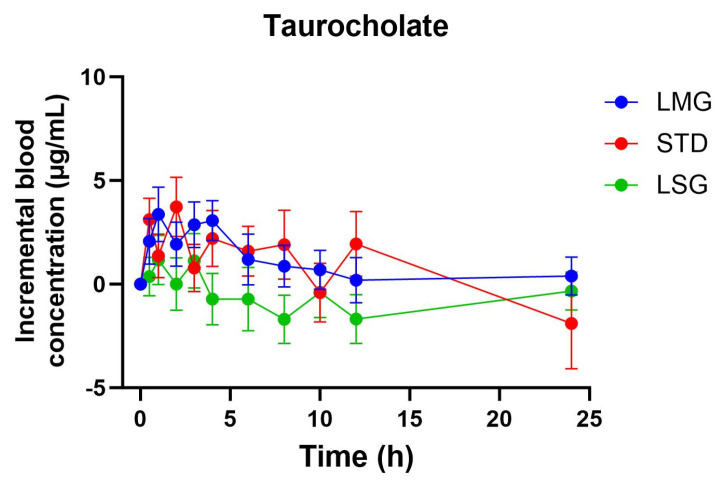
Blood concentration of taurocholate over 24 h. Data presented as mean ± SEM, *n* = 14. The iAUC and ΔC_max_ of LMG did not show significant differences compared to LSGs or STD using mixed-effects ANOVA with Tukey’s multiple comparisons test. LMG (LipoMicel^®^) and LSG (Liposomal) administered at a single dose of 300 mg GSH vs. STD (standard) at a higher dose of 500 mg GSH. Data have been normalized for dose.

Pharmacokinetic parameters for selected glutathione-related metabolites are summarized in Table 5, Table 6, Table 7, Table 8 and Table 9, visualized as graphs in Figure 7, Figure 8, Figure 9, Figure 10 and Figure 11, and presented as dose-normalized (300 mg equivalent). PK parameters for l-cystine, l-glutamate, and taurocholate showed no formulation-dependent differences in iAUC, ΔC_max_, or T_max_. For l-pyroglutamic acid (5-oxoproline), exposure metrics were not different across treatments (iAUC overall *p* = 0.322; ΔC_max_ overall *p* = 0.243), although T_max_ differed across treatments (overall *p* = 0.0153), with earlier peak timing for LMG (3.2 ± 1.1 h) versus STD (7.4 ± 1.2 h). In contrast, methionine showed formulation-dependent effects: iAUC was significantly higher with LMG than STD (149.9 ± 30.8 vs. 32.7 ± 28.3 µg·mL·h; *p* = 0.0151), corresponding to ~4.58-fold higher exposure, and ΔC_max_ was also higher (15.1 ± 2.4 vs. 4.6 ± 2.2 µg/mL; *p* = 0.0037), corresponding to ~3.28-fold higher peak response. Per mg dose-normalized results are provided in Supplementary Materials Tables S3–S7. Non-dose-corrected tables are presented in Supplementary Materials S3.

### 3.4. Safety—Blood Chemistry and Electrolytes

Several safety markers (as summarized in Table 10 and Table 11) were monitored in study participants over 30 days at a daily LMG administration of 600 mg/day. The results showed that LMG proved to be safe, with no significant changes in any of the tested blood markers.

### 3.5. Adverse Events

Other than two occurrences of moderate bloating and three occurrences of moderate nausea, only mild symptoms were reported (see Supplementary Tables S10 and S11). Gastrointestinal symptoms improved when the intervention was taken with food.

## 4. Discussion

By nature, an orally ingested health supplement is only efficacious if it can adequately cross the blood–gut barrier and enter into the circulatory system; in other words, it is only efficacious if it is also bioavailable [47]. The therapeutic efficacy of oral glutathione supplementation is well known to be limited by its low bioavailability due to instability and degradation issues occurring in the digestive tract. Moreover, the endogenous production of glutathione and the body’s stringent regulation of this compound add to the challenge in designing an efficacious oral formula [2].

Despite promising improvements to bioavailability in rodent models [20,21,48,49] relatively few controlled human studies have compared delivery-enhanced oral glutathione formulations. Accordingly, this trial evaluated whether a novel micellar delivery system (LipoMicel^®^; LMG) could increase systemic glutathione exposure relative to standard glutathione (STD) and a liposomal formulation (LSG) in healthy adults.

Previous studies found that standard oral glutathione might not effectively raise systemic levels in healthy individuals, primarily due to low bioavailability. For example, Allen et al. reported no significant differences in glutathione status or oxidative stress biomarkers after 4 weeks of oral glutathione supplementation at 500 mg twice daily [50]. Another study using high doses (3 g) of glutathione administered orally also found similarly unimpressive results [51]. To overcome low oral bioavailability or absorption barriers, formulation modifications can be a promising strategy in elevating oral glutathione bioavailability [2,44,52,53].

Our findings support the premise that delivery-enhanced formulation features can influence oral glutathione exposure. Both the liposomal and micellar (LipoMicel^®^) formulations increased baseline-adjusted whole-blood GSH relative to the standard preparation, with LipoMicel^®^ producing significantly greater incremental exposure over 24 h. These differences may relate to formulation-specific excipients and physicochemical properties (e.g., dispersion and carrier stability), as suggested by prior work—although these attributes were not directly characterized in the present study [54,55].

In this crossover PK study, compared with the standard formulation (STD, 500 mg), the micellar formulation (LMG, 300 mg) produced approximately 2.5-fold higher incremental exposure (iAUC) and 2.4-fold higher peak response (ΔC_max_), despite the lower administered dose (Table 3; Figure 4). Dose-normalized analyses (to a 300 mg equivalent) further supported greater exposure (iAUC was ~3.6-fold higher) with LMG (Table 2 and Table 4), while oxidized glutathione (GSSG) exposure did not differ between treatments.

One factor limiting large, sustained changes in circulating glutathione is homeostatic control of GSH and GSSG through rapid turnover, redistribution, and recycling [56,57,58]. Since GSSG exposure was unchanged across treatments, it suggests no significant increase in the oxidized glutathione pool under the study conditions. The GSH/GSSG ratio was higher with LMG versus STD, reflecting increased reduced glutathione levels without a corresponding rise in GSSG, and hence was consistent with a transient shift toward a more reduced glutathione redox couple. Collectively, these findings indicate that the LipoMicel^®^ formulation enhances systemic glutathione availability while preserving physiological redox control (i.e., without inducing oxidative burden).

In addition to glutathione and glutathione disulfide, this study evaluated a targeted panel of glutathione-related metabolites to provide complementary insight into sulfur amino acid handling, γ-glutamyl cycle activity, and hepatic sulfur utilization following oral supplementation. Across formulations, l-cystine and l-glutamate showed no significant differences in dose-normalized iAUC, ΔC_max_, or T_max_, consistent with tight regulation of these circulating pools. For 5-oxoproline (pyroglutamic acid), exposure did not differ between treatments; however, peak timing differed overall, with earlier T_max_ following LipoMicel^®^ administration. In contrast, methionine exhibited a formulation-dependent response, with significantly higher dose-normalized exposure and peak response after LipoMicel^®^ compared with the standard formulation. Because methionine is sensitive to nutritional and hepatic sulfur handling, this observation should be considered exploratory and may warrant confirmation in larger studies with expanded metabolite pathway coverage.

In summary, the biochemical changes following supplementation underscore the tight homeostatic regulation of glutathione and related sulfur metabolism. Notably, despite being administered at a lower dose (300 mg vs. 500 mg), LMG produced ~2.49-fold higher iAUC and ~2.43-fold higher ΔC_max_ versus the standard formulation, consistent with greater delivery efficiency. The lower LMG dose was selected a priori to evaluate whether formulation-dependent differences in delivery efficiency could enhance systemic glutathione exposure without increasing the administered amount. Dose-normalized analyses further supported greater exposure per unit dose with LMG (e.g., up to ~4-fold higher iAUC and C_max_ when expressed as a 300 mg equivalent; Table 2). Although nonlinear dose–exposure relationships cannot be excluded for an endogenously regulated analyte, the higher exposure observed with LipoMicel^®^ relative to both a higher-dose standard formulation and a dose-matched liposomal formulation supports a formulation-dependent contribution to systemic glutathione delivery. One potential contributor to these formulation-dependent differences may relate to the physicochemical characteristics of the delivery systems. While liposomal formulations typically consist of nanoscale particles—for example, Wei et al. reported that various liposome formulations of glutathione ranged in size from 250 to 600 nm [52]—LipoMicel^®^ formulations usually show particles in the micrometer range at approximately 50 µm [2,55]. While the smaller size of liposomes might suggest a finer dispersion, they could also be more fragile when subjected to the natural digestive actions, assisted by bile salts and lipase, leading to further instability [59]. The liposomes could consequently disintegrate, leading to a loss of the carrier effect. On the other hand, the many-fold larger LipoMicel^®^ could exhibit a better protective effect. With a bulkier topology, LipoMicel^®^ particles can withstand the same digestive actions without completely losing integrity, turning into smaller particles in the process. In fact, the LipoMicel^®^ strategy allows improvements in both dispersion and survival of carrier vehicles upon facing digestive actions. The survival of the finer LipoMicel^®^ vehicles can then facilitate uptake by the enterocytes rather than giving up the active ingredients to the intestinal fluid where enzymatic and pH-mediated degradation can occur [18]. However, further studies would be required to directly assess these mechanisms.

From a practical perspective, the improved bioavailability observed with LMG may offer advantages in terms of dosing efficiency and patient compliance. Achieving greater systemic exposure with a lower administered dose can reduce pill burden and support sustained supplementation, thereby enhancing the feasibility of long-term use.

Strengths of this study include quantification of glutathione and related analytes by LC-HRMS, whereas several prior studies relied on the glutathione reductase assay (also known as the 5,5′-dithiobis-(2-nitrobenzoic acid) (DTNB) assay) [18]. While rapid and sensitive, the reagent DTNB also reacts indiscriminately with other biological compounds that contain one or more thiol group(s). Some common compounds include cysteine, homocysteine, N-acetylcysteine, 1,4-dithioerythritol, protein thiols (e.g., albumin), coenzyme A, and cysteamine [60,61]. On the other hand, while the LC-HRMS assay used in this study may be more complex and costly, it offers unparalleled selectivity, precision, accuracy, and sensitivity [46,62]. Another aspect to note is that GSH and its metabolic partners are majorly present intracellularly, particularly within erythrocytes, rather than in plasma (i.e., extracellularly) [8,29]. This presents a challenge when analyzing bioavailability based on plasma content alone, as seen in previous PK studies. Therefore, quantifying GSH in whole blood, as seen in the current study, provides an integrated measure of both intracellular and extracellular pools, offering a more physiologically relevant index of systemic glutathione availability. The observed elevations in whole-blood GSH following LMG treatment indicate that such a formulation effectively increases circulating and cellular GSH levels. This suggests that the enhanced absorption achieved with the LipoMicel^®^ formulation was sufficient to raise total blood GSH despite rapid cellular uptake and turnover, reflecting improved systemic bioavailability. Overall, our study findings provide a comprehensive understanding of the metabolic impacts of glutathione supplementation and may suggest potential therapeutic benefits in managing oxidative stress-related conditions. Further research is warranted to elucidate the long-term effects of and optimal dosing strategies for glutathione supplementation in various clinical contexts.

### 4.1. Thirty-Day Safety Study

In addition to the pharmacokinetic investigation, we performed a 30-day study on GSH’s safety when formulated in a new, more bioavailable delivery system (i.e., LMG). Several markers that provide a comprehensive view of the supplement’s safety profile were monitored in participants, which included liver function tests (ALT, AST, ALP, etc.), renal function tests (serum creatinine, blood urea nitrogen (BUN), electrolytes (e.g., potassium, sodium)), and complete blood count (hemoglobin, hematocrit). Adverse events of a gastrointestinal nature were additionally recorded. No significant changes in any of these safety markers were detected. Thus, the LMG formulation proved to be safe and well tolerated in a healthy study population.

### 4.2. Limitations

This study has limitations. The modest sample size limits precision, particularly for secondary metabolite outcomes. While systemic GSH exposure increased, functional antioxidant endpoints (e.g., total antioxidant capacity) and longer-term clinical outcomes were not assessed. Because composite antioxidant assays reflect extracellular antioxidant pools and are influenced by multiple circulating factors beyond glutathione, future studies should incorporate functional endpoints alongside PK and expanded sulfur-metabolism panels to clarify physiological relevance and optimal dosing.

## 5. Conclusions

In this randomized crossover study, the LipoMicel^®^ micellar formulation (LMG) produced significantly higher baseline-adjusted systemic glutathione exposure than standard glutathione despite a lower administered dose. Compared with STD (500 mg), LMG at a lower dose (300 mg) increased iAUC and ΔC_max_ by ~2.49-fold and ~2.43-fold, respectively, and when dose-normalized (to a 300 mg equivalent), incremental systemic exposure and peak levels were up to ~4-fold higher, supporting greater absorption per unit dose. Oxidized glutathione (GSSG) exposure was unchanged, while the GSH/GSSG ratio was higher with LMG, indicating increased reduced glutathione availability without a measurable increase in the oxidized pool (GSSG). These findings support LipoMicel^®^ as a promising delivery approach for oral glutathione and warrant confirmation in larger studies incorporating functional redox endpoints and longer-term clinical outcomes.

## Figures and Tables

**Table 1:** Study population demographic data (*n* = 14).

Baseline Characteristics	Mean ± SD
Age (years)	37.2 ± 12.1
Weight (kg)	63.9 ± 10.8
Height (cm)	167.6 ± 7.8
BMI (kg/m^2^)	22.6 ± 2.8

BMI: Body mass index.

**Table 2:** Dose-normalized pharmacokinetic parameters of glutathione (GSH) following single-dose administration of three formulations (mean ± SEM; *n* = 14).

	LMG	LSG	STD	Overall *p*-Value	*p*-Value LMG vs. STD
T_max_ (h)	13.6 ± 2.8	15.8 ± 2.4	9.9 ± 2.2	0.4595	0.7319
iAUC_0–24_ (µg·mL^−1^·h)	1287.5 ± 179.0	645.5 ± 165.0	359.6 ± 108.0	0.0018 *	0.0040 *
∆C_max_ (µg/mL)	103.9 ± 11.8	45.5 ± 10.7	25.7 ± 6.9	0.0003 *	0.0003 *

Data are expressed as the mean ± SEM; *n* = 14. Overall *p*-value reflects the comparison among the three treatments (LMG, LSG, STD) using mixed ANOVA. Pairwise *p*-values (LMG vs. STD) are from post hoc testing following ANOVA: Tukey’s multiple comparisons test for iAUC_0–24_, ∆C_max_, and T_max_. Dose-corrected analysis of LMG (LipoMicel^®^) and LSG (Liposomal) administered at a dose of 300 mg GSH vs. STD (standard) administered at a higher dose of 500 mg GSH; dose-normalized (300 mg equivalent). Asterisks (*) denote statistically significant differences (*p* ≤ 0.05).

**Table 3:** Pharmacokinetic parameters of glutathione (GSH) following single-dose administration of three formulations (mean ± SEM; *n* = 14).

	LMG	LSG	STD	Overall *p*-Value	*p*-Value LMG vs. STD
T_max_ (h)	13.6 ± 2.8	15.8 ± 2.4	9.9 ± 2.2	0.4595	0.7319
iAUC_0–24_ (µg·mL^−1^·h)	1287.5 ± 179.0	645.5 ± 165.0	517.8 ± 180.0	0.0067 *	0.0064 *
∆C_max_ (µg/mL)	103.9 ± 11.8	45.5 ± 10.7	42.8 ± 11.5	0.0005 *	0.0003 *

Data showing results from actual administered doses, not corrected. Data are expressed as the mean ± SEM; *n* = 14. Overall *p*-value reflects the comparison among the three treatments (LMG, LSG, STD) using mixed-effects ANOVA. Pairwise *p*-values (LMG vs. STD) are from post hoc testing following ANOVA: Tukey’s multiple comparisons test for iAUC_0–24_, ∆C_max_, and T_max_. Doses: LMG = 300 mg, LSG = 300 mg, STD = 500 mg. Asterisks (*) denote statistically significant differences (*p* ≤ 0.05).

**Table 4:** Dose-normalized pharmacokinetic parameters of GSSG following single-dose administration of three formulations (mean ± SEM; *n* = 14).

	LMG	LSG	STD	Overall *p*-Value	*p*-Value LMG vs. STD
T_max_ (h)	1.8 ± 0.8	3.9 ± 1.7	2.8 ± 0.9	0.4651	0.5410
iAUC_0–24_ (µg·mL^−1^·h)	136.4 ± 63.4	121.5 ± 57.6	47.27 ± 69.3	0.583	0.941
∆C_max_ (µg/mL)	54.9 ± 24.9	25.5 ± 22.9	6.9 ± 27.2	0.422	0.578

Data are expressed as the mean ± SEM; *n* = 14. Overall *p*-values reflect comparison among the three treatments (LMG, LSG, STD) using mixed-effects ANOVA. Pairwise *p*-values (LMG vs. STD) used Tukey’s multiple comparisons test; data are dose-normalized (300 mg equivalent).

**Table 5:** Dose-normalized pharmacokinetic parameters of l-cystine following single-dose administration of three formulations (mean ± SEM; *n* = 14).

	LMG	LSG	STD	Overall *p*-Value	*p*-Value LMG vs. STD
T_max_ (h)	7.4 ± 1.8	9.5 ± 2.5	10.3 ± 2.3	0.6631	0.7384
iAUC_0–24_ (µg·mL^−1^·h)	10.5 ± 2.9	16.2 ± 3.2	11.4 ± 3.1	0.374	0.854
∆C_max_ (µg/mL)	1.0 ± 0.2	1.5 ± 0.2	1.2 ± 0.2	0.225	0.612

Data are expressed as the mean ± SEM; *n* = 14. Overall *p*-values reflect global comparison among the three treatments (LMG, LSG, STD) using mixed-effects ANOVA. Pairwise *p*-values (LMG vs. STD) used Tukey’s multiple comparisons test; data have been normalized for a dose of 300 mg.

**Table 6:** Dose-normalized pharmacokinetic parameters of l-glutamate following single-dose administration of three formulations (mean ± SEM; *n* = 14).

	LMG	LSG	STD	Overall *p*-Value	*p*-Value LMG vs. STD
T_max_ (h)	5.1 ± 1.3	5.9 ± 1.2	4.8 ± 1.1	0.9034	0.9925
iAUC_0–24_ (µg·mL^−1^·h)	5.8 ± 7.9	24.0 ± 7.7	6.4 ± 7.7	0.0828	0.9944
∆C_max_ (µg/mL)	1.5 ± 0.5	2.1 ± 0.5	1.3 ± 0.5	0.5009	0.8249

Data are expressed as the mean ± SEM; *n* = 14. Overall *p*-values reflect global comparison among the three treatments (LMG, LSG, STD) using mixed-effects analysis. Pairwise *p*-values (LMG vs. STD) used Tukey’s multiple comparisons test. Doses: LMG = 300 mg, LSG = 300 mg, STD = 500 mg. Data have been normalized for a dose of 300 mg.

**Table 7:** Dose-normalized pharmacokinetic parameters of L-pyroglutamic acid following single-dose administration of three formulations (mean ± SEM; *n* = 14).

	LMG	LSG	STD	Overall *p*-Value	*p*-Value LMG vs. STD
T_max_ (h)	3.2 ± 1.1	6.4 ± 0.9	7.4 ± 1.2	0.0153 *	0.0887
iAUC_0–24_ (µg·mL^−1^·h)	19.8 ± 8.6	35.4 ± 9.1	27.1 ± 9.0	0.322	0.647
∆C_max_ (µg/mL)	2.5 ± 0.7	4.3 ± 0.8	3.8 ± 0.7	0.243	0.636

Data are expressed as the mean ± SEM; *n* = 14. Overall *p*-values reflect global comparison among the three treatments (LMG, LSG, STD) using mixed-effects analysis. Pairwise *p*-values (LMG vs. STD) used Tukey’s multiple comparisons test; data have been normalized for a dose of 300 mg. Asterisks (*) denote statistically significant differences (*p* ≤ 0.05).

**Table 8:** Dose-normalized pharmacokinetic parameters of methionine following single-dose administration of three formulations (mean ± SEM; *n* = 14).

	LMG	LSG	STD	Overall *p*-Value	*p*-Value LMG vs. STD
T_max_ (h)	8.6 ± 1.1	3.8 ± 1.2	5.2 ± 1.4	0.0906	0.2341
iAUC_0–24_ (µg·mL^−1^·h)	149.9 ± 30.8	75.5 ± 32.2	32.7 ± 28.3	0.0280 *	0.0151 *
∆C_max_ (µg/mL)	15.1 ± 2.4	7.6 ± 2.5	4.6 ± 2.2	0.0092 *	0.0037 *

Data are expressed as the mean ± SEM; *n* = 14. Overall *p*-values reflect global comparison among the three treatments (LMG, LSG, STD) using mixed-effects analysis. Pairwise *p*-values (LMG vs. STD) used Tukey’s multiple comparisons test. Data have been normalized for a dose of 300 mg. Asterisks (*) denote statistically significant differences (*p* ≤ 0.05).

**Table 9:** Dose-normalized pharmacokinetic parameters of taurocholate following single-dose administration of three formulations (mean ± SEM; *n* = 14).

	LMG	LSG	STD	Overall *p*-Value	*p*-Value LMG vs. STD
T_max_ (h)	4.7 ± 1.1	3.2 ± 1.8	4.1 ± 1.5	0.2079	0.7594
iAUC_0–24_ (µg·mL^−1^·h)	47.1 ± 12.5	31.4 ± 12.5	57.5 ± 13.7	0.3820	0.7430
∆C_max_ (µg/mL)	5.6 ± 1.0	3.6 ±1.0	5.1 ± 1.1	0.3500	0.7570

Data are expressed as the mean ± SEM; *n* = 14. Overall *p*-values reflect global comparison among the three treatments (LMG, LSG, STD) using mixed-effects analysis. Pairwise *p*-values (LMG vs. STD) used Tukey’s multiple comparisons test. Data have been normalized for a dose of 300 mg.

**Table 10:** Blood chemistry and electrolyte data for male participants over 30 days. N = 6 males.

Males	Results (Mean ± SD)			*p*-Value	Normal Range
	Week 0	Week 2	Week 4		
ALT (U/L)	25.0 ± 10.47	23.5 ± 9.07	23.33 ± 9.97	0.785	9–50
AST (U/L)	20.83 ± 5.71	20.5 ± 3.83	21.67 ± 5.28	0.537	15–40
Total Bilirubin (µmol/L)	20.5 ± 8.02	13.17 ± 5.81	21.33 ± 10.73	0.135	3.4–21.0
Creatinine (µmol/L)	82.67 ± 4.68	80.67 ± 8.98	84.17 ± 10.15	0.266	44.0–97.0
Glucose (mmol/L)	5.33 ± 0.26	5.1 ± 0.30	5.05 ± 0.31	0.227	3.89–6.11
Total Cholesterol (mmol/L)	5.48 ± 1.09	5.22 ± 1.01	5.54 ± 1.54	0.089	2.0–5.19
Sodium (mmol/L)	139.67 ± 0.81	139.17 ± 1.72	138.8 ± 0.75	0.383	135–147
Potassium (mmol/L)	4.37 ± 0.31	4.17 ± 0.32	4.05 ± 0.41	0.113	3.4–5.3
ALP(U/L)	74.83 ± 15.78	75.83 ± 17.39	74.83 ± 18.64	0.830	35–120
BUN (mmol/L)	5.35 ± 1.06	4.72 ± 1.32	5.07 ± 1.17	0.292	2.5–8.2
GFR (mL/min/1.73 m^2^)	99.81 ± 7.63	99.80 ± 8.76	95.63 ± 8.56	0.159	≥60
CRP (mg/L)	0.88 ± 0.43	0.64 ± 0.19	0.78 ± 0.39	0.146	≤5.0
RBC (10^12^/L)	5.09 ± 0.39	5.01 ± 0.40	5.18 ± 0.26	0.111	3.5–5.5
WBC (10^9^/L)	5.97 ± 0.85	6.07 ± 0.82	5.18 ± 1.10	0.669	4.0–10.0
Hemoglobin (g/L)	151.50 ± 10.82	149.67 ± 11.14	150.83 ± 8.11	0.546	115–155
Hematocrit (L/L)	0.45 ± 0.04	0.45 ± 0.03	0.46 ± 0.02	0.222	0.35–0.45
LDL (mmol/L)	3.62 ± 0.97	3.24 ± 0.91	3.47 ± 0.98	0.275	1.50–3.40
HDL (mmol/L)	1.31 ± 0.24	1.22 ± 0.22	1.30 ± 0.34	0.315	>1.19
Triglycerides (mmol/L)	1.21 ± 0.52	1.68 ± 1.11	1.7 ± 0.97	0.248	≤2.21

*p*-values are calculated from one-way repeated-measures ANOVA (within-subject factor: time). No significant time effects were detected (all *p* > 0.05). Reference ranges shown for clinical context.

**Table 11:** Blood chemistry and electrolyte data for female participants over 30 days. N = 6 females.

Females	Results (Mean ± SD)			*p*-Value	Normal Range
	Week 0	Week 2	Week 4		
ALT (U/L)	11.5 ± 1.7	15.6 ± 5.5	12.8 ± 3.3	0.279	7–40
AST (U/L)	19.5 ± 3.4	21.8 ± 1.5	19.6 ± 2.3	0.171	13–35
Total Bilirubin (µmol/L)	11.2 ± 5.1	13.0 ± 10.7	13.0 ± 7.3	0.684	3.4–21.0
Creatinine (µmol/L)	69.6 ± 4.5	67.2 ± 5.0	68.8 ± 4.1	0.442	35–80
Glucose (mmol/L)	5.1 ± 0.68	4.74 ± 0.31	5.05 ± 0.31	0.444	3.89–6.11
Total Cholesterol (mmol/L)	4.78 ± 1.25	4.59 ± 1.29	4.82 ± 1.5	0.826	2.0–5.19
Sodium (mmol/L)	137.6 ± 0.89	136.33 ± 1.75	137.6 ± 0.89	0.480	135–147
Potassium (mmol/L)	4.58 ± 0.51	4.2 ± 0.2	4.32 ± 0.08	0.294	3.4–5.3
ALP(U/L)	66.40 ± 5.50	64.80 ± 8.04	71.60 ± 4.28	0.271	35–120
BUN (mmol/L)	4.84 ± 1.20	4.34 ± 1.36	5.04 ± 1.59	0.310	24–155
GFR (mL/min/1.73 m^2^)	92.25 ± 8.54	98.50 ± 9.68	93.50 ± 10.50	0.190	≥60
CRP (mg/L)	1.13 ± 0.71	0.67 ± 0.29	1.70 ± 1.15	0.417	≤5.0
RBC (10^12^/L)	4.71 ± 0.17	4.68 ± 0.34	4.67 ± 0.23	0.841	3.50–5.00
WBC (10^9^/L)	6.10 ± 1.02	5.78 ± 1.30	5.96 ± 0.99	0.656	4.0–10.0
Hemoglobin (g/L)	136.60 ± 6.69	135.60 ± 11.92	134.0 ± 8.37	0.762	115–155
Hematocrit (L/L)	0.42 ± 0.02	0.42 ± 0.03	0.42 ± 0.03	0.838	0.35–0.45
LDL (mmol/L)	2.88 ± 0.97	2.73 ± 1.01	2.97 ± 1.28	0.585	1.50–3.40
HDL (mmol/L)	1.58 ± 0.38	1.56 ± 0.36	1.50 ± 0.33	0.699	>1.19
Triglycerides (mmol/L)	0.69 ± 0.14	0.90 ± 0.19	0.80 ± 0.16	0.067	≤2.21

*p*-values are calculated from one-way repeated-measures ANOVA (within-subject factor: time). No significant time effects were detected (all *p* > 0.05). Reference ranges shown for clinical context.

## Data Availability

Data and/or statistical analyses are available upon request.

## Supplementary Materials

The following supporting information can be downloaded at https://www.mdpi.com/article/10.3390/antiox15030354/s1.

## Abbreviations

The following abbreviations are used in this manuscript:

ALT	Alanine aminotransferase
ALP	Alkaline phosphatase
AST	Aspartate aminotransferase
AUC	Area under curve
BUN	Blood urea nitrogen
C_max_	Peak plasma concentration
CRP	C-reactive protein
EDTA	Ethylenediaminetetraacetic acid
eGFR	Estimated glomerular filtration rate
GGT	γ-glutamyl transferase
GSH	Glutathione
GSSG	Glutathione disulfide
HDL	High-density lipoprotein
LC-HRMS	Liquid chromatography–high-resolution mass spectrometry
LC-MS	Liquid chromatography–mass spectrometry
LDL	Low-density lipoprotein
LMG	LipoMicel^®^ glutathione formulation
LSG	Liposomal glutathione formulation
MRTi	Mean residence time
STD	Standard glutathione
T_max_	Time at which maximum concentration is reached
WBC	White blood cell
UHPLC	Ultra-high-performance liquid chromatography

## References

[1] Meister A., Anderson M.E. (1983). Glutathione. Annu. Rev. Biochem..

[2] Schmitt B., Vicenzi M., Garrel C., Denis F.M. (2015). Effects of N-Acetylcysteine, Oral Glutathione (GSH) and a Novel Sublingual Form of GSH on Oxidative Stress Markers: A Comparative Crossover Study. Redox Biol..

[3] Sen C.K. (1998). Redox Signaling and the Emerging Therapeutic Potential of Thiol Antioxidants. Biochem. Pharmacol..

[4] Meister A. (1994). Glutathione, Ascorbate, and Cellular Protection1. Cancer Res..

[5] Jones D.P., Mody V.C., Carlson J.L., Lynn M.J., Sternberg P. (2002). Redox Analysis of Human Plasma Allows Separation of Pro-Oxidant Events of Aging from Decline in Antioxidant Defenses. Free Radic. Biol. Med..

[6] Polonikov A. (2020). Endogenous Deficiency of Glutathione as the Most Likely Cause of Serious Manifestations and Death in COVID-19 Patients. ACS Infect. Dis..

[7] Ballatori N., Krance S.M., Notenboom S., Shi S., Tieu K., Hammond C.L. (2009). Glutathione Dysregulation and the Etiology and Progression of Human Diseases. Biol. Chem..

[8] Wu G., Fang Y.-Z., Yang S., Lupton J.R., Turner N.D. (2004). Glutathione Metabolism and Its Implications for Health. J. Nutr..

[9] Horowitz R.I., Freeman P.R., Bruzzese J. (2020). Efficacy of Glutathione Therapy in Relieving Dyspnea Associated with COVID-19 Pneumonia: A Report of 2 Cases. Respir. Med. Case Rep..

[10] Guloyan V., Oganesian B., Baghdasaryan N., Yeh C., Singh M., Guilford F., Ting Y.-S., Venketaraman V. (2020). Glutathione Supplementation as an Adjunctive Therapy in COVID-19. Antioxidants.

[11] Silvagno F., Vernone A., Pescarmona G.P. (2020). The Role of Glutathione in Protecting against the Severe Inflammatory Response Triggered by COVID-19. Antioxidants.

[12] Gamcsik M.P., Kasibhatla M.S., Teeter S.D., Colvin O.M. (2012). Glutathione Levels in Human Tumors. Biomarkers.

[13] Townsend D.M., Tew K.D., Tapiero H. (2003). The Importance of Glutathione in Human Disease. Biomed. Pharmacother..

[14] Sharma D.K., Sharma P. (2022). Augmented Glutathione Absorption from Oral Mucosa and Its Effect on Skin Pigmentation: A Clinical Review. Clin. Cosmet. Investig. Dermatol..

[15] Dewan B., Shinde S. (2022). Glutathione an Effective Adjuvant Therapy for Acute Respiratory Distress Syndrome Associated with COVID-19 Infection. J. Adv. Med. Med. Res..

[16] Marianetti M., Pinna S., Venuti A., Liguri G. (2022). Olive Polyphenols and Bioavailable Glutathione: Promising Results in Patients Diagnosed with Mild Alzheimer’s Disease. Alzheimer’s Dement. Transl. Res. Clin. Interv..

[17] Sechi G., Deledda M.G., Bua G., Satta W.M., Deiana G.A., Pes G.M., Rosati G. (1996). Reduced Intravenous Glutathione in the Treatment of Early Parkinson’s Disease. Prog. Neuro-Psychopharmacol. Biol. Psychiatry.

[18] Wei T., Thakur S.S., Liu M., Wen J. (2022). Oral Delivery of Glutathione: Antioxidant Function, Barriers and Strategies. Acta Mater. Medica.

[19] Zhang H., Jay Forman H., Choi J., Sies H., Packer L. (2005). γ-Glutamyl Transpeptidase in Glutathione Biosynthesis. Methods in Enzymology.

[20] Crankshaw D.L., Briggs J.E., Vince R., Nagasawa H.T. (2021). An Orally Bioavailable (Mice) Prodrug of Glutathione. Antioxidants.

[21] Byeon J.C., Lee S.-E., Kim T.-H., Ahn J.B., Kim D.-H., Choi J.-S., Park J.-S. (2019). Design of Novel Proliposome Formulation for Antioxidant Peptide, Glutathione with Enhanced Oral Bioavailability and Stability. Drug Deliv..

[22] Sinha R., Sinha I., Calcagnotto A., Trushin N., Haley J.S., Schell T.D., Richie J.P. (2018). Oral Supplementation with Liposomal Glutathione Elevates Body Stores of Glutathione and Markers of Immune Function. Eur. J. Clin. Nutr..

[23] Atkuri K.R., Mantovani J.J., Herzenberg L.A., Herzenberg L.A. (2007). N-Acetylcysteine—A Safe Antidote for Cysteine/Glutathione Deficiency. Curr. Opin. Pharmacol..

[24] De Rosa S.C., Zaretsky M.D., Dubs J.G., Roederer M., Anderson M., Green A., Mitra D., Watanabe N., Nakamura H., Tjioe I. (2000). N-Acetylcysteine Replenishes Glutathione in HIV Infection. Eur. J. Clin. Investig..

[25] Raftos J.E., Whillier S., Chapman B.E., Kuchel P.W. (2007). Kinetics of Uptake and Deacetylation of N-Acetylcysteine by Human Erythrocytes. Int. J. Biochem. Cell Biol..

[26] Lushchak V.I. (2012). Glutathione Homeostasis and Functions: Potential Targets for Medical Interventions. J. Amino Acids.

[27] Arrick B.A., Nathan C. (1984). Glutathione Metabolism as a Determinant of Therapeutic Efficacy: A Review. Cancer Res..

[28] Zilmer M., Soomets U., Rehema A., Langel Ü. (2005). The Glutathione System as an Attractive Therapeutic Target. Drug Des. Rev.-Online.

[29] Vašková J., Kočan L., Vaško L., Perjési P. (2023). Glutathione-Related Enzymes and Proteins: A Review. Molecules.

[30] Greenberg D. (2012). Metabolism of Sulfur Compounds.

[31] Robaczewska J., Kedziora-Kornatowska K., Kozakiewicz M., Zary-Sikorska E., Pawluk H., Pawliszak W., Kedziora J. (2016). Role of Glutathione Metabolism and Glutathione-Related Antioxidant Defense Systems in Hypertension. J. Physiol. Pharmacol..

[32] Zarka M.H., Bridge W.J. (2017). Oral Administration of γ-Glutamylcysteine Increases Intracellular Glutathione Levels above Homeostasis in a Randomised Human Trial Pilot Study. Redox Biol..

[33] Novelli A., Bianchetti A. (2022). Glutathione: Pharmacological Aspects and Implications for Clinical Use. Geriatr. Care.

[34] Banerjee R. (2012). Redox Outside the Box: Linking Extracellular Redox Remodeling with Intracellular Redox Metabolism. J. Biol. Chem..

[35] Banjac A., Perisic T., Sato H., Seiler A., Bannai S., Weiss N., Kölle P., Tschoep K., Issels R.D., Daniel P.T. (2008). The Cystine/Cysteine Cycle: A Redox Cycle Regulating Susceptibility versus Resistance to Cell Death. Oncogene.

[36] Jyotsana N., Ta K.T., DelGiorno K.E. (2022). The Role of Cystine/Glutamate Antiporter SLC7A11/xCT in the Pathophysiology of Cancer. Front. Oncol..

[37] Emmett M. (2014). Acetaminophen Toxicity and 5-Oxoproline (Pyroglutamic Acid): A Tale of Two Cycles, One an ATP-Depleting Futile Cycle and the Other a Useful Cycle. Clin. J. Am. Soc. Nephrol..

[38] Geenen S., Guallar-Hoyas C., Michopoulos F., Kenna J.G., Kolaja K.L., Westerhoff H.V., Thomas P., Wilson I.D. (2011). HPLC–MS/MS Methods for the Quantitative Analysis of 5-Oxoproline (Pyroglutamate) in Rat Plasma and Hepatic Cell Line Culture Medium. J. Pharm. Biomed. Anal..

[39] Mannucci L., Pastore A., Rizzo C., Piemonte F., Rizzoni G., Emma F. (2006). Impaired Activity of the γ-Glutamyl Cycle in Nephropathic Cystinosis Fibroblasts. Pediatr. Res..

[40] Mayatepek E. (1999). 5-Oxoprolinuria in Patients with and without Defects in the -Glutamyl Cycle. Eur. J. Pediatr..

[41] Stipanuk M.H. (2020). Metabolism of Sulfur-Containing Amino Acids: How the Body Copes with Excess Methionine, Cysteine, and Sulfide. J. Nutr..

[42] Miyazaki T., Ueda H., Ikegami T., Honda A. (2023). Upregulation of Taurine Biosynthesis and Bile Acid Conjugation with Taurine through FXR in a Mouse Model with Human-like Bile Acid Composition. Metabolites.

[43] Flori L., Veneziano S., Martelli A., Piragine E., Calderone V. (2025). Transsulfuration Pathway Products and H2S-Donors in Hyperhomocysteinemia: Potential Strategies Beyond Folic Acid. Int. J. Mol. Sci..

[44] Buonocore D., Grosini M., Giardina S., Michelotti A., Carrabetta M., Seneci A., Verri M., Dossena M., Marzatico F. (2015). Bioavailability Study of an Innovative Orobuccal Formulation of Glutathione. Oxidative Med. Cell. Longev..

[45] Ibi A., Chang C., Kuo Y.C., Zhang Y., Do P., Du M., Roh Y.S., Gahler R., Hardy M., Solnier J. (2025). Comparative Pharmacokinetics and Safety of a Micellar Chrysin–Quercetin–Rutin Formulation: A Randomized Crossover Trial. Antioxidants.

[46] Squellerio I., Caruso D., Porro B., Veglia F., Tremoli E., Cavalca V. (2012). Direct Glutathione Quantification in Human Blood by LC–MS/MS: Comparison with HPLC with Electrochemical Detection. J. Pharm. Biomed. Anal..

[47] Toutain P.L., Bousquet-Mélou A. (2004). Bioavailability and Its Assessment. J. Vet. Pharmacol. Ther..

[48] Liu Y., Hyde A.S., Simpson M.A., Barycki J.J. (2014). Emerging Regulatory Paradigms in Glutathione Metabolism. Adv. Cancer Res..

[49] Aboubakr E.M., Mohammed H.A., Hassan A.S., Mohamed H.B., Dosoky M.I.E., Ahmad A.M. (2022). Glutathione-Loaded Non-Ionic Surfactant Niosomes: A New Approach to Improve Oral Bioavailability and Hepatoprotective Efficacy of Glutathione. Nanotechnol. Rev..

[50] Allen J., Bradley R.D. (2011). Effects of Oral Glutathione Supplementation on Systemic Oxidative Stress Biomarkers in Human Volunteers. J. Altern. Complement. Med..

[51] Witschi A., Reddy S., Stofer B., Lauterburg B.H. (1992). The Systemic Availability of Oral Glutathione. Eur. J. Clin. Pharmacol..

[52] Bruggeman B.K., Storo K.E., Fair H.M., Wommack A.J., Carriker C.R., Smoliga J.M. (2019). The Absorptive Effects of Orobuccal Non-Liposomal Nano-Sized Glutathione on Blood Glutathione Parameters in Healthy Individuals: A Pilot Study. PLoS ONE.

[53] Richie J., Nichenametla S., Neidig W., Calcagnotto A., Haley J., Schell T., Muscat J. (2014). Randomized Controlled Trial of Oral Glutathione Supplementation on Body Stores of Glutathione. Eur. J. Nutr..

[54] Ibi A., Chang C., Zhang Y., Kuo Y.C., Du M., Roh K., Gahler R., Solnier J. (2025). An in Vitro Investigation on the Physicochemical Properties of Different Quercetin Formulations. J. Complement. Integr. Med..

[55] Du M., Chang C., Zhang X., Zhang Y., Radford M.J., Gahler R.J., Kuo Y.C., Wood S., Solnier J. (2023). Designing Vitamin D3 Formulations: An In Vitro Investigation Using a Novel Micellar Delivery System. Nutraceuticals.

[56] Cesarini L., Grignaffini F., Alisi A., Pastore A. (2024). Alterations in Glutathione Redox Homeostasis in Metabolic Dysfunction-Associated Fatty Liver Disease: A Systematic Review. Antioxidants.

[57] Tandon R., Tandon A. (2024). Unraveling the Multifaceted Role of Glutathione in Sepsis: A Comprehensive Review. Cureus.

[58] Dzah C.S., Zhang H., Gobe V., Asante-Donyinah D., Duan Y. (2024). Anti- and pro-Oxidant Properties of Polyphenols and Their Role in Modulating Glutathione Synthesis, Activity and Cellular Redox Potential: Potential Synergies for Disease Management. Adv. Redox Res..

[59] Cui J., Wen Z., Zhang W., Wu W. (2022). Recent Advances in Oral Peptide or Protein-Based Drug Liposomes. Pharmaceuticals.

[60] Özyürek M., Baki S., Güngör N., Karademir Çelik S., Güçlü K., Apak R. (2012). Determination of Biothiols by a Novel On-Line HPLC-DTNB Assay with Post-Column Detection. Anal. Chim. Acta.

[61] Balcerczyk A., Grzelak A., Janaszewska A., Jakubowski W., Koziol S., Marszalek M., Rychlik B., Soszynski M., Biliński T., Bartosz G. (2003). Thiols as Major Determinants of the Total Antioxidant Capacity. BioFactors.

[62] Thiel A., Weishaupt A.-K., Nicolai M.M., Lossow K., Kipp A., Schwerdtle T., Bornhorst J. (2023). Simultaneous Quantitation of Oxidized and Reduced Glutathione via LC-MS/MS to Study the Redox State and Drug-Mediated Modulation in Cells, Worms and Animal Tissue. J. Chromatogr. B.

