# Supplementary material for: A Targeted Metabolomic Assessment of Oral Glutathione Bioavailability and Safety in Humans: A Randomized Crossover Clinical Trial
Source: Antioxidants (Basel). 2026 Mar 11;15(3):354. doi: 10.3390/antiox15030354 (PMC13023597; doi:10.3390/antiox15030354)
Supplement: Supplementary file 1 [file antioxidants-15-00354-s001.zip › Supplementary Materials S1 Per mg Dose-Corrected PK Tables.pdf]

Supplementary Materials S1: Per mg Dose-Corrected PK Tables

GSH

Table S1. Dose-normalized pharmacokinetic parameters of GSH per mg of administered dose.

	LMG per mg	LSG per mg	STD per mg	Overall *p* value	*p* value of LMG vs STD
$iAUC_{0\text{-}24}$ (μg·mL^{-1}·h)	4.3 ± 0.8	2.2 ± 0.5	2.0 ± 0.7	0.0001 *	0.0049 *
ΔC_{max} (μg/mL)	0.35 ± 0.1	0.16 ± 0.02	0.15 ± 0.03	0.0060 *	0.0458 *

Data are expressed as the mean ± SEM; *n* = 14. Overall *p*-values reflect comparison of dose-normalized data (per mg) among the three treatments (LMG, LSG, STD) using Mixed-effects ANOVA. Pairwise *p*-values (LMG vs STD) used Tukey's multiple comparisons test; actual doses administered: LMG = 300 mg, LSG = 300 mg, STD = 500 mg. Asterisks (*) denote statistically significant differences ($p \leq 0.05$).

GSSG

Table S2. Dose-normalized pharmacokinetic parameters of GSSG per mg of administered dose.

	LMG per mg	LSG per mg	STD per mg	Overall *p* value	*P* value LMG vs STD
$iAUC_{0\text{-}24}$ (μg·mL^{-1}·h)	0.5 ± 0.2	0.4 ± 0.2	0.3 ± 0.23	0.2503	0.4175
ΔC_{max} (μg/mL)	0.2 ± 0.1	0.1 ± 0.1	0.02 ± 0.2	0.3351	0.3041

Data are expressed as the mean ± SEM; *n* = 14. Overall *p*-values reflect global comparison of dose-normalized data (per mg) among the three treatments (LMG, LSG, STD) using Mixed-effects ANOVA. Pairwise *p*-values (LMG vs STD) used Tukey's multiple comparisons test; actual doses administered: LMG = 300 mg, LSG = 300 mg, STD = 500 mg. Asterisks (*) denote statistically significant differences ($p \leq 0.05$).

L-cystine

Table S3. Dose-normalized pharmacokinetic parameters of L-cystine ($AUC_{0–24}$ or C_{max} by administered dose (per mg)).

	LMG per mg	LSG per mg	STD per mg	Overall *p* value	*P* value LMG vs STD
$iAUC_{0\text{-}24}$ (μg·mL^{-1}·h)	0.04 ± 0.01	0.05 ± 0.01	0.03 ± 0.01	0.1781	0.9630

ΔC_{max} (μg/mL)	0.003 ± 0.001	0.005 ± 0.001	0.003 ± 0.001	0.1071	0.8784

Data are expressed as the mean ± SEM; *n* = 14. Overall *p*-values reflect global comparison of dose-normalized data (per mg) among the three treatments (LMG, LSG, STD) using Mixed-effects ANOVA. Pairwise *p*-values (LMG vs STD) used Tukey's multiple comparisons test; actual doses administered: LMG = 300 mg, LSG = 300 mg, STD = 500 mg. Asterisks (*) denote statistically significant differences ($p \leq 0.05$).

L-glutamate

Table S4. Dose-normalized pharmacokinetic parameters of L-glutamate ($AUC_0–_{24}$ or C_{max} by administered dose (per mg)).

	LMG per mg	LSG per mg	STD per mg	Overall *p* value	*P* value LMG vs STD
$iAUC_{0-24}$ ($\mu g \cdot mL^{-1} \cdot h$)	0.02 ± 0.03	0.08 ± 0.03	0.02 ± 0.03	0.1353	0.0913
ΔC_{max} (μg/mL)	0.005 ± 0.002	0.007 ± 0.002	0.005 ± 0.002	0.3805	0.9730

Data are expressed as the mean ± SEM; *n* = 14. Overall *p*-values reflect global comparison of dose-normalized data (per mg) among the three treatments (LMG, LSG, STD) using mixed effects analysis. Pairwise *p*-values (LMG vs STD) used Tukey's multiple comparisons test; actual doses administered: LMG = 300 mg, LSG = 300 mg, STD = 500 mg.

L-pyroglutamic acid

Table S5. Dose-normalized pharmacokinetic parameters of L-pyroglutamic acid ($AUC_0–_{24}$ or C_{max} by administered dose (per mg).

	LMG per mg	LSG per mg	STD per mg	Overall *p* value	*P* value LMG vs STD
$iAUC_{0-24}$ ($\mu g \cdot mL^{-1} \cdot h$)	0.07 ± 0.03	0.12 ± 0.03	0.08 ± 0.03	0.2863	0.3856
ΔC_{max} (μg/mL)	0.01 ± 0.002	0.01 ± 0.002	0.02 ± 0.002	0.3028	0.4420

Data are expressed as the mean ± SEM; *n* = 14. Overall *p*-values reflect global comparison of dose-normalized data (per mg) among the three treatments (LMG, LSG, STD) using mixed effects analysis. Pairwise

p-values (LMG vs STD) used Tukey's multiple comparisons test; actual doses administered: LMG = 300 mg, LSG = 300 mg, STD = 500 mg.

Methionine

Table S6. Dose-normalized pharmacokinetic parameters of methionine ($AUC_{0–24}$ or C_{max} by administered dose (per mg)).

	LMG per mg	LSG per mg	STD per mg	Overall *p* value	*P* value LMG vs STD
$iAUC_{0-24}$ ($\mu g \cdot mL^{-1} \cdot h$)	0.5 ± 0.1	0.3 ± 0.1	0.2 ± 0.08	0.0175 *	0.0355 *
ΔC_{max} (μg/mL)	0.05 ± 0.01	0.03 ± 0.01	0.02 ± 0.006	0.0350 *	0.0642

Data are expressed as the mean ± SEM; *n* = 14. Overall *p*-values reflect global comparison of dose-normalized data (per mg) among the three treatments (LMG, LSG, STD) using mixed effects analysis. Pairwise *p*-values (LMG vs STD) used Tukey's multiple comparisons test; actual doses administered: LMG = 300 mg, LSG = 300 mg, STD = 500 mg. Asterisks (*) denote statistically significant differences ($p \leq 0.05$).

Taurocholate

Table S7. Dose-normalized pharmacokinetic parameters of taurocholate ($AUC_{0–24}$ or C_{max} by administered dose (per mg)).

	LMG per mg	LSG per mg	STD per mg	Overall *p* value	*P* value LMG vs STD
$iAUC_{0-24}$ ($\mu g \cdot mL^{-1} \cdot h$)	0.16 ± 0.04	0.10 ± 0.04	0.20 ± 0.05	0.3557	0.4969
ΔC_{max} (μg/mL)	0.02 ± 0.003	0.01 ± 0.003	0.02 ± 0.003	0.7230	0.9281

Data are expressed as the mean ± SEM; *n* = 14. Overall *p*-values reflect global comparison of dose-normalized data (per mg) among the three treatments (LMG, LSG, STD) using mixed effects analysis. Pairwise *p*-values (LMG vs STD) used Tukey's multiple comparisons test; actual doses administered: LMG = 300 mg, LSG = 300 mg, STD = 500 mg. Asterisks (*) denote statistically significant differences ($p \leq 0.05$).
