# Supplementary material for: A Targeted Metabolomic Assessment of Oral Glutathione Bioavailability and Safety in Humans: A Randomized Crossover Clinical Trial
Source: Antioxidants (Basel). 2026 Mar 11;15(3):354. doi: 10.3390/antiox15030354 (PMC13023597; doi:10.3390/antiox15030354)
Supplement: Supplementary file 1 [file antioxidants-15-00354-s001.zip › Supplementary Materials S2 Exposure Ratios, Baseline Comparisons and Safety Data.pdf]

Supplementary Materials S2: Exposure Ratios, Baseline Comparisons and Safety Data

Exposure Ratios

Table S8. Ratio of LMG to STD exposure for glutathione-related metabolites based on geometric mean AUC_{0-24} and C_{max} values

	**Raw data**		**Normalized data**	
	AUC	C_{max}	AUC	C_{max}
**GSH**	1.813	1.768	3.022	2.947
**GSSG**	0.680	0.870	1.134	1.449
**L-Cystine**	0.829	0.750	1.382	1.250
**L-Glutamate**	0.994	0.838	1.657	1.397
**L-Pyroglutamic acid**	0.715	0.648	1.191	1.080
**methionine**	1.447	1.105	2.411	1.842
**Taurocholate**	0.917	0.838	1.528	1.397

Baseline Comparisons

Table S9. Baseline (Time 0) concentrations of glutathione and metabolites by treatment.

**Analyte**	**LMG**	**STD**	**LSG**	***p* (LMG vs STD)**	***p* (LMG vs LSG)**	***p* (STD vs LSG)**
GSH	91.15 ± 13.73	70.47 ± 9.88	85.92 ± 9.84	0.0829	0.9784	0.9188
GSSG	19.58 ± 4.24	24.43 ± 4.04	23.82 ± 4.76	0.9422	0.8156	0.1253
L-cystine	3.36 ± 0.40	4.18 ± 0.37	3.73 ± 0.41	0.4503	0.9853	0.863
L-glutamate	6.91 ± 1.38	6.75 ± 0.88	7.50 ± 1.82	0.472	>0.9999	0.9626
L-pyroglutamic acid	8.54 ± 0.84	9.95 ± 0.81	9.77 ± 0.46	0.3273	0.7991	0.9826
Methionine	20.98 ± 2.78	23.87 ± 2.14	18.40 ± 2.19	0.5376	0.6386	0.315
Taurocholate	19.31 ± 1.06	18.95 ± 2.40	20.92 ± 1.10	0.9972	0.78	0.7633

Concentrations are expressed in units of µg/mL as mean ± SEM for all analytes; *n* = 14. Tukey's multiple comparisons test was applied post-hoc for pair-wise *p*-values using a mixed-effects model with fixed effects

for analyte, treatment, and analyte×treatment. No treatment effect was observed at baseline (Treatment p = 0.7801; Analyte×Treatment p = 0.6239). All Tukey-adjusted pairwise p-values within each analyte were > 0.05.

Safety Summary

Table S10. Number of participants reporting adverse events during the safety segment of the study.

	**Week 1**	**Week 2**	**Week 3**	**Week 4**
Participants reporting AE, *n* (%)	1 (7%)	1 (7%)	2 (13%)	1 (7%)

N = total number of participants = 15. n = number of participants reporting AE per week. n/N, the weekly fraction of participants who'd reported AE, is represented as a percentage in parentheses (%).

Table S11. Adverse events listed by preferred terms and corresponding severities.

**Preferred Terms**	**Mild Events**	**Moderate Events**
Bloating	3	2
Constipation	2	
Diarrhea	1	
Pain or cramps	2	
Nausea	2	3

Numerical values indicate the number of occurrences of a specific AE, at either the "mild" or "moderate" severity. No serious or life-threatening AEs occurred throughout the study.
