# Supplementary material for: A Targeted Metabolomic Assessment of Oral Glutathione Bioavailability and Safety in Humans: A Randomized Crossover Clinical Trial
Source: Antioxidants (Basel). 2026 Mar 11;15(3):354. doi: 10.3390/antiox15030354 (PMC13023597; doi:10.3390/antiox15030354)
Supplement: Supplementary file 1 [file antioxidants-15-00354-s001.zip › Supplementary Materials S3 Non-Dose-Corrected PK Figures and Tables.pdf]

Supplementary Materials S3: Non-Dose-Corrected PK Figures and Tables

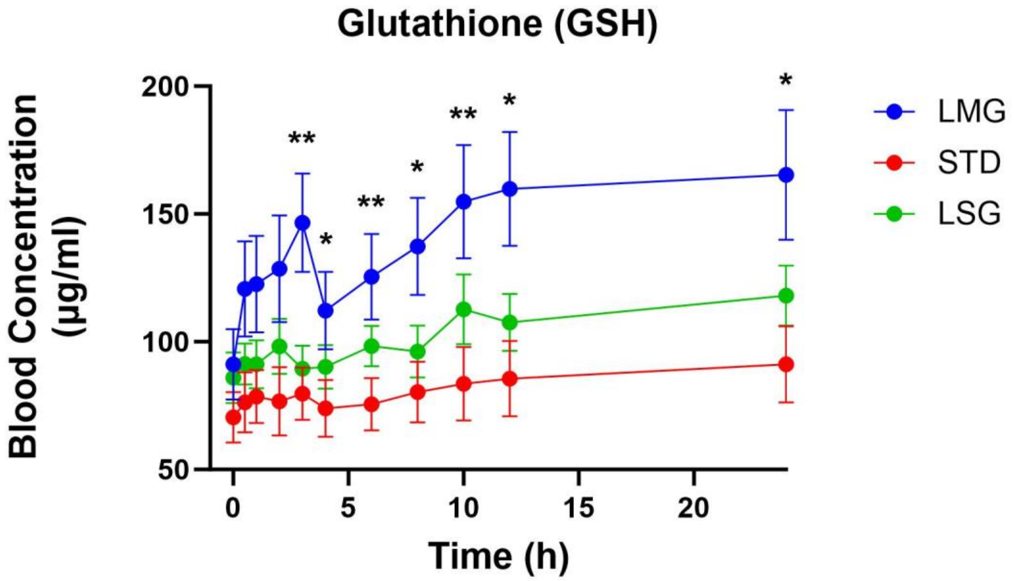

Figure S1. Blood concentration trend of Glutathione (GSH) over 24hrs. Data presented as Mean ± SEM, *n* = 14. Data were analyzed using Mixed-effects ANOVA with Tukey's multiple comparisons test.; LMG (LipoMicel) and LSG (Liposomal) administered at a single dose of 300 mg GSH vs STD (standard) administered at a higher dose of 500 mg GSH. Significant differences between LMG and STD with $p \leq 0.05$ are marked with a single asterisk (*) whereas differences with $p \leq 0.01$ are marked with double asterisk (**).

Table S12. Pharmacokinetic parameters of glutathione (GSH) following single-dose administration of three formulations (mean ± SEM; *n* = 14).

	**LMG**	**LSG**	**STD**	**Overall *p* value**	***p* value LMG vs STD**
**AUC_{0-24} (µg·mL^{-1}·h)**	3355.6 ± 480.7	2429.2± 245.5	1840.3 ± 288.5	0.0157*	0.0123*
**C_{max} (µg/mL)**	195.1 ± 23.3	131.4 ± 10.5	107.4 ± 12.7	0.0017*	0.0016*
**$T_{1/2}$ (h)**	299.7 ± 252.9	85.9 ± 34.6	101.7 ± 44.9	0.7564	0.9701
**T_{max} (h)**	13.6 ± 2.8	15.8 ± 2.4	9.9 ± 2.2	0.4595	0.7319
**MRTi (h)**	435.3 ± 363.5	127.1 ± 49.6	148.2 ± 64.2	0.7571	0.9650

Data are expressed as the mean ± SEM; *n* = 14. Overall *p* value reflects the global comparison among the three treatments (LMG, LSG, STD) using one-way ANOVA. Pairwise *p*-values (LMG vs STD) are from post-hoc testing following ANOVA: Tukey's multiple comparisons test for AUC_{0-24} , C_{max}, and T_{max}, and Dunnett T3 for $T_{1/2}$ and MRTi (used where SEM differed). Data have not been normalized for dose; Doses: LMG = 300 mg, LSG = 300 mg, STD = 500 mg. Asterisks (*) denote statistically significant differences ($p \leq 0.05$).

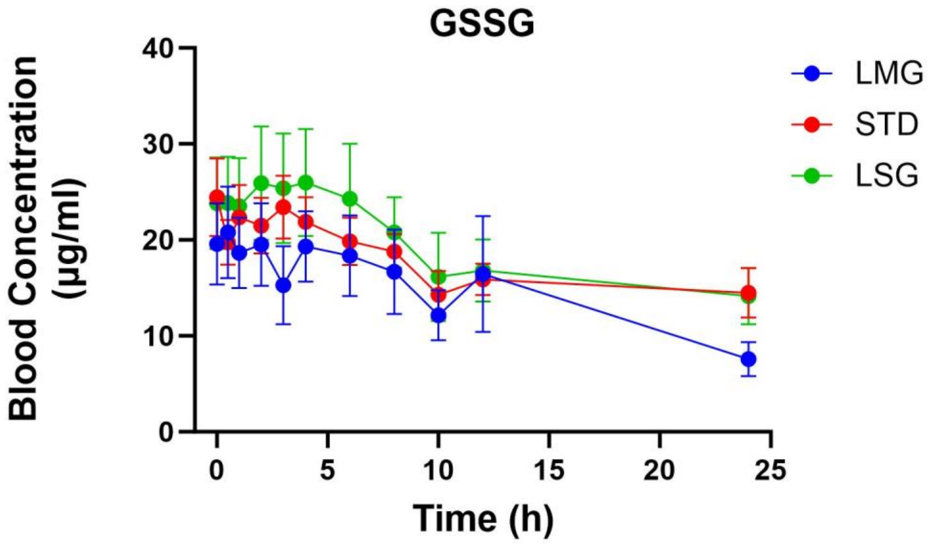

Figure S2. Blood concentrations trend of GSSG over 24hrs. Data presented as Mean ± SEM, *n* = 14. The AUC or C_{max} of LMG did not show significant difference compared to LSGs or STD using Mixed-effects ANOVA with Tukey's multiple comparisons test.; LMG (LipoMicel) and LSG (Liposomal) administered at a single dose of 300 mg GSH vs STD (standard) at a higher dose of 500 mg GSH.

Table S13. Pharmacokinetic parameters of GSSG following single-dose administration of three formulations (mean ± SEM; *n* = 14).

	**LMG**	**LSG**	**STD**	**Overall *p* value**	***P* value LMG vs STD**
**AUC_{0-24} ($\mu g \cdot mL^{-1} \cdot h$)**	311.1 ± 60.4	428.1± 75.9	397.1 ± 41.3	0.4147	0.6489
**C_{max} (µg/mL)**	36.1 ± 15.1	31.1 ± 6.5	27.9 ± 3.8	0.8976	0.8896
**$T_{1/2}$ (h)**	22.6 ± 2.6	27.2 ± 3.7	73.6 ± 31.5	0.1830	0.1830
**T_{max} (h)**	1.8 ± 0.8	3.9 ± 1.7	2.8 ± 0.9	0.4651	0.5410
**MRTi (h)**	33.3 ± 3.6	40.4 ± 5.3	107.9 ± 45.3	0.0807	0.0799

Data are expressed as the mean ± SEM; *n* = 14. Overall *p*-values reflect global comparison among the three treatments (LMG, LSG, STD) using one-way ANOVA. Pairwise *p*-values (LMG vs STD) used Tukey's multiple comparisons test; Doses: LMG = 300 mg, LSG = 300 mg, STD = 500 mg.

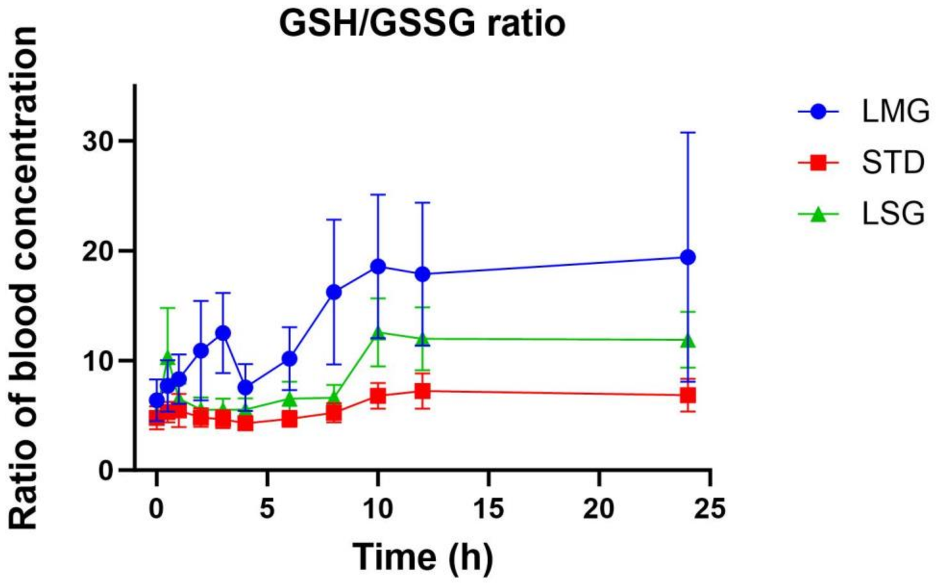

Figure S3. The ratio of GSH to GSSG in blood concentration is demonstrated. LMG shows a significant difference in GSH/GSSG ratio compared to STD (p=0.001), but no significant difference when compared to LSG (p=0.1478) using Mixed-effects ANOVA with Tukey's multiple comparisons test.

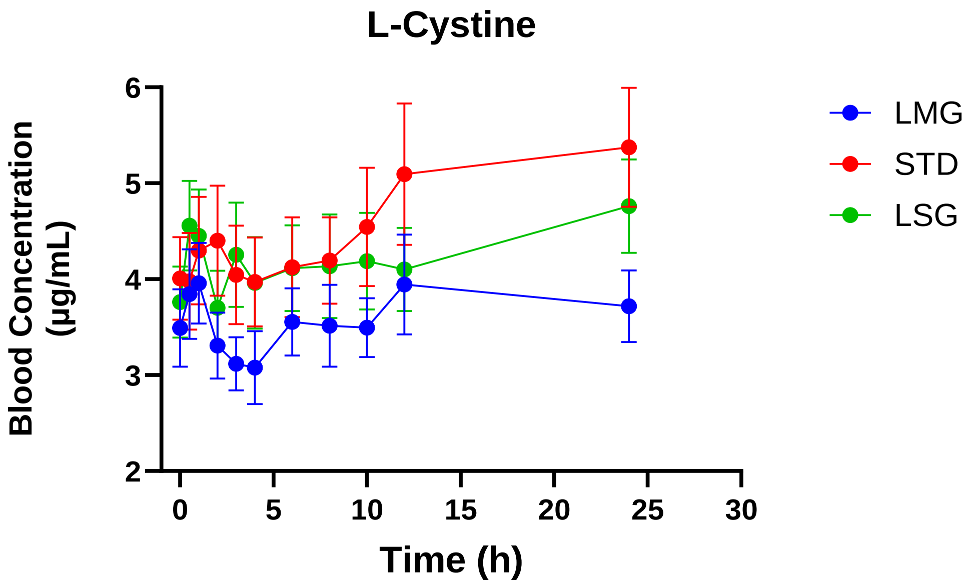

Figure S4. Blood concentration of L-cystine over 24hrs. Data presented as Mean ± SEM, n = 14. The AUC or C_{max} of LMG did not show significant difference compared to LSGs or STD using Mixed-effects ANOVA with Tukey's multiple comparisons test.; LMG (LipoMicel) and LSG (Liposomal) administered at a single dose of 300 mg GSH vs STD (standard) at a higher dose of 500 mg GSH.

Table S14. Pharmacokinetic parameters of L-cystine following single-dose administration of three formulations (mean ± SEM; *n* = 14).

	LMG	LSG	STD	Overall *p* value	*P* value LMG vs STD
**AUC_{0-24} (μg·mL^{-1}·h)**	83.9 ± 9.5	100.5± 10.6	104.2 ± 14.3	0.3867	0.5699
**C_{max} (μg/mL)**	4.5 ± 0.4	5.3 ± 0.6	5.9 ± 0.6	0.1747	0.2363
**$T_{1/2}$ (h)**	219.9 ± 82.3	105.0 ± 24.2	771.3 ± 555.2	0.1033	0.3199
**T_{max} (h)**	7.4 ± 1.8	9.5 ± 2.5	10.3 ± 2.3	0.6631	0.7384
**MRTi (h)**	319.2 ± 119.1	155.4 ± 35.6	1115.5 ± 801.2	0.1069	0.3198

Data are expressed as the mean ± SEM; *n* = 14. Overall *p*-values reflect global comparison among the three treatments (LMG, LSG, STD) using one-way ANOVA. Pairwise *p*-values (LMG vs STD) used Tukey's multiple comparisons test; Doses: LMG = 300 mg, LSG = 300 mg, STD = 500 mg.

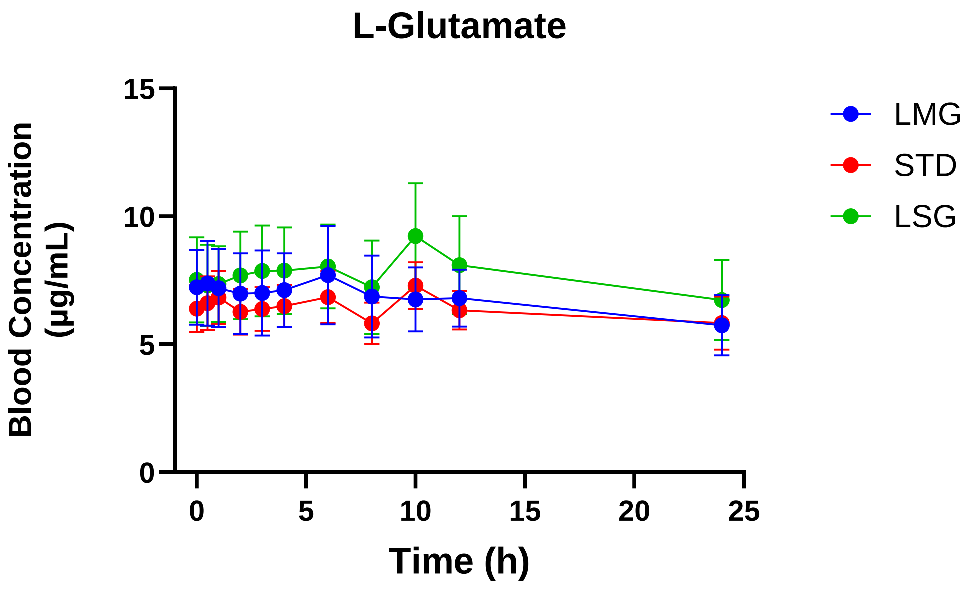

Figure S5. Blood concentration of L-glutamate over 24hrs. Data presented as Mean ± SEM, *n* = 14. The AUC or C_{max} of LMG did not show significant difference compared to LSGs or STD using Mixed-effects ANOVA with Tukey's multiple comparisons test.; LMG (LipoMicel) and LSG (Liposomal) administered at a single dose of 300 mg GSH vs STD (standard) at a higher dose of 500 mg GSH.

Table S15. Pharmacokinetic parameters of L-glutamate following single-dose administration of three formulations (mean ± SEM; *n* = 14).

	LMG	LSG	STD	Overall *p* value	*P* value LMG vs STD
**AUC_{0-24} (μg·mL^{-1}·h)**	151.5 ± 33.3	176.8 ± 39.9	139.7 ± 20.5	0.8223	0.9998
**C_{max} (μg/mL)**	8.4 ± 2.1	9.6 ± 2.0	8.6 ± 1.1	0.7677	0.8193

**$T_{1/2}$ (h)**	45.8 ± 11.1	46.6 ± 7.4	48.3 ± 13.3	0.9442	0.9994
**T_{max} (h)**	5.1 ± 1.3	5.9 ± 1.2	4.8 ± 1.1	0.9034	0.9925
**MRTi (h)**	67.6 ± 16.0	68.9 ± 10.8	70.9 ± 19.1	0.9434	0.9990

Data are expressed as the mean ± SEM; *n* = 14 Overall *p*-values reflect global comparison among the three treatments (LMG, LSG, STD) using mixed effects analysis. Pairwise *p*-values (LMG vs STD) used Tukey's multiple comparisons test; Doses: LMG = 300 mg, LSG = 300 mg, STD = 500 mg.

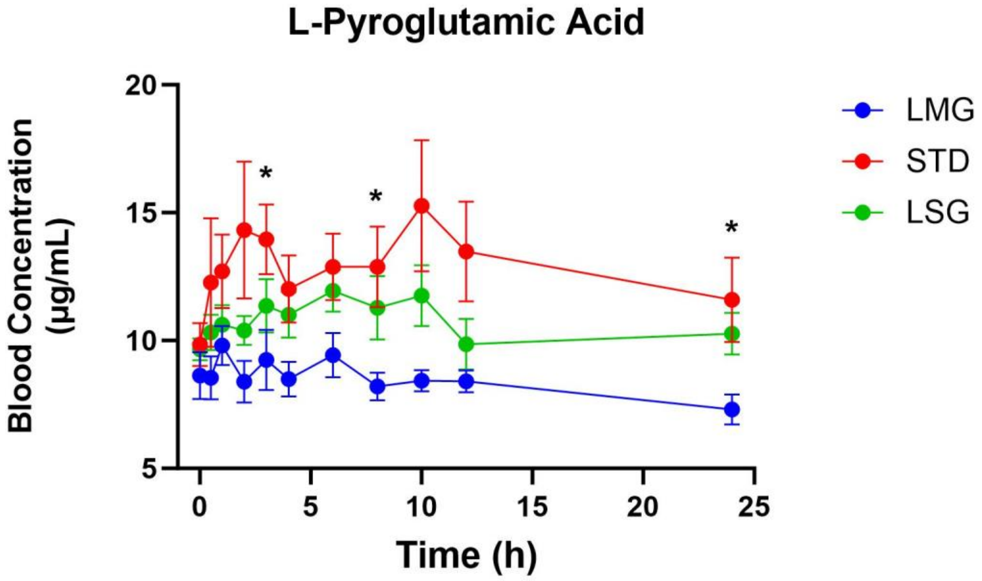

Figure S6. Blood concentration of L-pyroglutamic acid over 24hrs. Data presented as Mean ± SEM, n = 14. The AUC and C_{max} of LMG showed significant difference compared to LSGs or STD using Mixed-effects ANOVA with Tukey's multiple comparisons test.; LMG (LipoMicel) and LSG (Liposomal) administered at a single dose of 300 mg GSH vs STD (standard) at a higher dose of 500 mg GSH. Significant differences between LMG and STD with $p \leq 0.05$ are marked with a single asterisk (*) whereas differences with $p \leq 0.01$ are marked with double asterisk (**).

Table S16. Pharmacokinetic parameters of L-pyroglutamic acid following single-dose administration of three formulations (mean ± SEM; *n* = 14).

	**LMG**	**LSG**	**STD**	**Overall *p* value**	***P* value LMG vs STD**
**$AUC_{0\text{-}24}$ ($\mu g \cdot mL^{-1} \cdot h$)**	187.1 ± 10.8	246.1 ± 21.4	286.9 ± 39.8	0.0497 *	0.0164 *
**C_{max} (µg/mL)**	11.1 ± 1.1	13.8 ± 0.9	18.2 ± 2.6	0.0309 *	0.0448 *
**$T_{1/2}$ (h)**	55.1 ± 14.5	133.5 ± 80.3	42.7 ± 17.9	0.4124	0.8570
**T_{max} (h)**	3.2 ± 1.1	6.4 ± 0.9	7.4 ± 1.2	0.0153 *	0.0887
**MRTi (h)**	80.9 ± 20.9	193.9 ± 115.9	63.4 ± 25.7	0.4250	0.8740

Data are expressed as the mean ± SEM; *n* = 14. Overall *p*-values reflect global comparison among the three treatments (LMG, LSG, STD) using mixed effects analysis. Pairwise *p*-values (LMG vs STD) used Tukey's multiple comparisons test; Doses: LMG = 300 mg, LSG = 300 mg, STD = 500 mg.

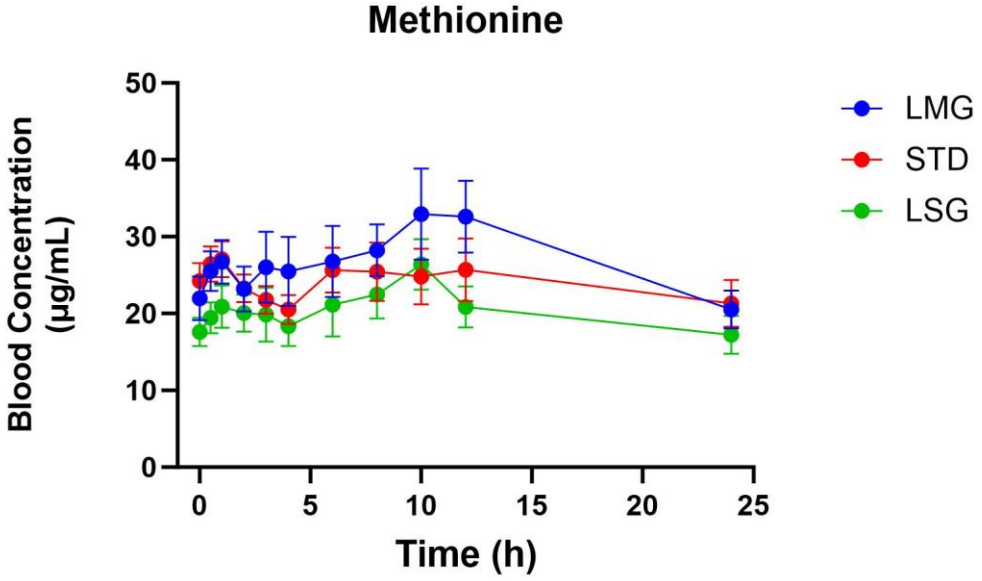

Figure S7. Blood concentration of methionine over 24hrs. Data presented as Mean ± SEM, n = 14. The AUC or C_{max} of LMG did not show significant difference compared to LSGs or STD using Mixed-effects ANOVA with Tukey's multiple comparisons test; LMG (LipoMicel) and LSG (Liposomal) administered at a single dose of 300 mg GSH vs STD (standard) at a higher dose of 500 mg GSH.

Table S17. Pharmacokinetic parameters of methionine following single-dose administration of three formulations (mean ± SEM; *n* = 14).

	**LMG**	**LSG**	**STD**	**Overall *p* value**	***P* value LMG vs STD**
**AUC_{0-24} (µg·mL^{-1}·h)**	638.3 ± 79.7	479.2 ± 64.8	483.1 ± 71.4	0.1437	0.2756
**C_{max} (µg/mL)**	36.7 ± 4.9	25.3 ± 3.5	32.5 ± 2.8	0.0672	0.8514
**$T_{1/2}$ (h)**	42.3 ± 1.2	50.1 ± 13.5	52.9 ± 13.5	0.6397	0.9082
**T_{max} (h)**	8.6 ± 1.1	3.8 ± 1.2	5.2 ± 1.4	0.0906	0.2341
**MRTi (h)**	62.8 ± 16.9	73.6 ± 19.3	76.8 ± 19.5	0.6873	0.9454

Data are expressed as the mean ± SEM; *n* = 14. Overall *p*-values reflect global comparison among the three treatments (LMG, LSG, STD) using mixed effects analysis. Pairwise *p*-values (LMG vs STD) used Tukey's multiple comparisons test; Doses: LMG = 300 mg, LSG = 300 mg, STD = 500 mg.

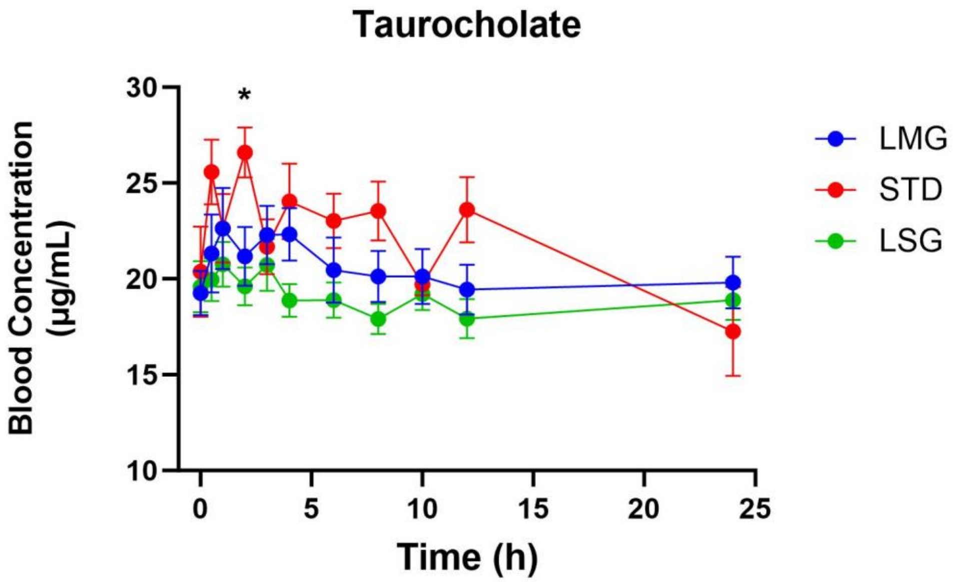

Figure S8. Blood concentration of taurocholate over 24hrs. Data presented as Mean ± SEM, n = 14. The C_{max}, T_{max} and MRTi of LMG showed significant difference compared to LSGs or STD using Mixed-effects ANOVA with Tukey's multiple comparisons test.; LMG (LipoMicel) and LSG (Liposomal) administered at a single dose of 300 mg GSH vs STD (standard) at a higher dose of 500 mg GSH. Significant differences between LMG and STD with $p \leq 0.05$ are marked with a single asterisk (*) whereas differences with $p \leq 0.01$ are marked with double asterisk (**).

Table S18. Pharmacokinetic parameters of taurocholate following single-dose administration of three formulations (mean ± SEM; *n* = 14).

	**LMG**	**LSG**	**STD**	**Overall *p* value**	***P* value LMG vs STD**
**AUC_{0-24} (µg·mL^{-1}·h)**	468.7 ± 86.1	433.5 ± 27.3	500.9 ± 33.3	0.4392	0.7875
**C_{max} (µg/mL)**	24.8 ± 2.0	23.2 ± 1.1	28.9 ± 1.2	0.0440 *	0.3403
**$T_{1/2}$ (h)**	214.9 ± 14.5	81.1 ± 14.8	42.5 ± 11.6	0.0432 *	0.1841
**T_{max} (h)**	4.7 ± 1.1	3.2 ± 1.8	4.1 ± 1.5	0.2079	0.7594
**MRTi (h)**	311.4 ± 123.9	118.5 ± 21.5	62.6 ± 16.3	0.0450 *	0.2041

Data are expressed as the mean ± SEM; *n* = 14. Overall *p*-values reflect global comparison among the three treatments (LMG, LSG, STD) using mixed-effects analysis. Pairwise *p*-values (LMG vs STD) used Tukey's multiple comparisons test; Doses: LMG = 300 mg, LSG = 300 mg, STD = 500 mg.
