# Supplementary material for: A Targeted Metabolomic Assessment of Oral Glutathione Bioavailability and Safety in Humans: A Randomized Crossover Clinical Trial
Source: Antioxidants (Basel). 2026 Mar 11;15(3):354. doi: 10.3390/antiox15030354 (PMC13023597; doi:10.3390/antiox15030354)
Supplement: Supplementary file 1 [file antioxidants-15-00354-s001.zip › Supplementary Materials S4 Addendum Carryover Assessment.pdf]

Supplementary Material S4 - Addendum

Carryover / accumulation assessment

To evaluate potential carryover or dose accumulation across crossover periods, we compared pre-dose (t=0) whole-blood GSH concentrations by study phase (Phase I, II, III) after assigning each participant's period-specific treatment using the randomization schedule (A=LMG, B=STD, C=LSG). Baseline GSH values were not systematically elevated in later phases. In paired within-participant comparisons, Phase II vs Phase I showed a small non-significant increase (mean difference = +18.1 μg/mL; paired t-test *p*=0.182; Wilcoxon *p*=0.232; n=10), while Phase III vs Phase I showed no increase (mean difference = −6.7 μg/mL; paired t-test *p*=0.752; Wilcoxon *p*=0.791; n=12). A linear mixed-effects model with participant as a random intercept showed no overall phase effect on baseline GSH (likelihood-ratio $\chi^2(2)=0.233$, *p*=0.890). Together with the absence of baseline treatment differences in Table S2, these results support no evidence of systematic carryover/accumulation influencing treatment comparisons.

Table S19. Pre-dose (t=0) whole-blood GSH by phase (period) and paired phase comparisons.
Phase baselines reflect the treatment assigned in that phase (A=LMG, B=STD, C=LSG) per the randomization schedule; t=0 values were taken from the corresponding treatment-period in the raw GSH dataset.

Comparison (paired within participant)	Mean difference (μg/mL) (Later – Earlier)	SD of difference	Paired t-test *p*	Wilcoxon *p*
**Phase II – Phase I**	+18.1	39.6	0.182	0.232
**Phase III – Phase I**	−6.7	71.7	0.752	0.791

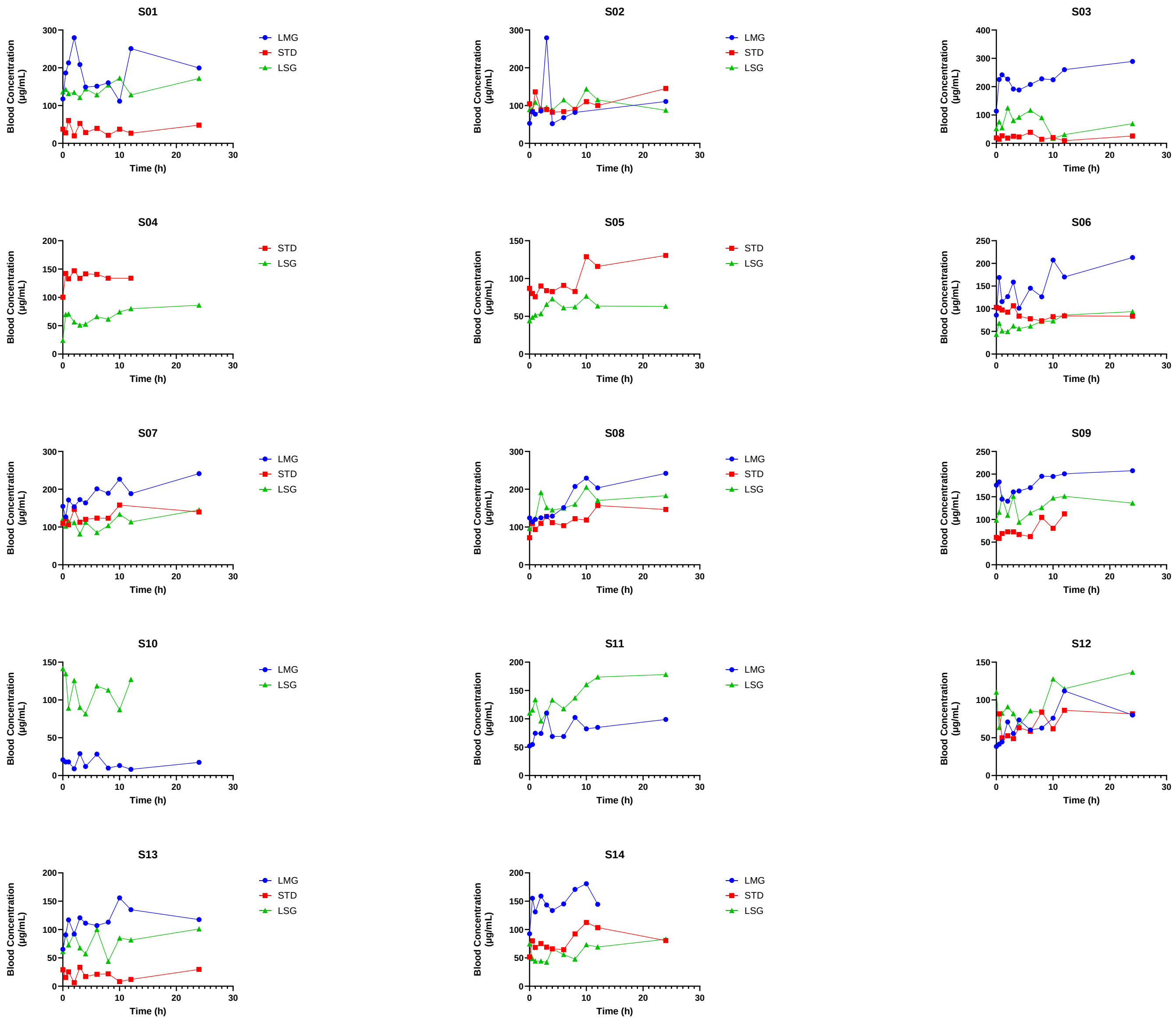

Figure S9. Individual whole-blood GSH concentration–time profiles by participant (raw concentrations). For each participant, concentration–time curves are shown for the available treatments (LMG, STD, LSG) over 0–24 h; curves are overlaid within participant to facilitate visual assessment of pre-dose (t=0) comparability and potential carryover/accumulation between crossover periods. All participants completed all phases and samples were analyzed; occasional missing curves reflect analyte-specific non-quantifiable results, not missed study visits. Across participants, there is no consistent pre-dose elevation in later periods, and baseline (t=0) concentrations did not differ by treatment in a mixed-effects analysis (Table S2) nor show systematic elevation by phase (Table S5). Because GSH is endogenous and baseline variability across periods is expected, primary PK endpoints were analyzed as baseline-adjusted measures (incremental $AUC_{0\text{-}24}$ and ΔC_{max}).

Supplementary Table S20. Blood chemistry and electrolyte parameters at 0 h and 24 h

Blood chemistry and electrolyte parameters measured at baseline (0 h, pre-dose) and 24 h post-dose for each treatment (LMG, STD, LSG). Values are mean ± SD; n indicates participants with paired 0 h and 24 h values available for that analyte and treatment. Within-treatment changes were assessed using two-sided paired t-tests on Δ(24 h − 0 h). Differences in mean change between treatments were evaluated using an Ordinary Least Squares (OLS) model with participant as a blocking factor (change ~ participant + treatment). Values reported as below the quantification limit (“<x”) were imputed as x/2 prior to analysis (e.g., “<0.30” → 0.15).

Parameter	p (Treatment effect on Δ)	LMG n	LMG 0h	LMG 24h	LMG Δ(24-0)	LMG p (paired)	STD n	STD 0h	STD 24h	STD Δ(24-0)	STD p (paired)	LSG n	LSG 0h	LSG 24h	LSG Δ(24-0)	LSG p (paired)
A/G	0.1647	14	1.43 ± 0.15	1.44 ± 0.16	0.01 ± 0.08	0.5176	13	1.37 ± 0.14	1.42 ± 0.15	0.05 ± 0.08	0.0455	13	1.41 ± 0.15	1.41 ± 0.14	0.00 ± 0.06	0.8542
ALP (U/L)	0.789	14	45.43 ± 9.97	45.43 ± 10.26	0.00 ± 3.66	1.0	13	44.46 ± 8.42	43.23 ± 11.17	-1.23 ± 10.24	0.6724	13	47.54 ± 11.38	45.54 ± 9.12	-2.00 ± 5.37	0.2041
ALT (U/L)	0.5846	14	26.00 ± 17.69	28.57 ± 16.28	2.57 ± 8.61	0.284	13	22.46 ± 4.52	22.46 ± 7.29	0.00 ± 6.67	1.0	13	27.38 ± 15.35	27.62 ± 14.03	0.23 ± 2.80	0.7717
AMY (U/L)	0.4132	14	66.50 ± 19.33	68.21 ± 18.63	1.71 ± 7.22	0.3902	13	68.77 ± 32.85	71.77 ± 22.13	3.00 ± 29.79	0.7228	13	68.08 ± 26.91	65.62 ± 18.96	-2.46 ± 13.26	0.5159
AST (U/L)	0.4352	14	23.21 ± 7.21	24.29 ± 6.26	1.07 ± 5.43	0.4732	13	20.65 ± 8.39	22.08 ± 5.07	1.42 ± 7.38	0.5003	13	22.69 ± 7.12	21.19 ± 9.48	-1.50 ± 5.42	0.3377
BUN (mmol/L)	0.0232	14	4.75 ± 1.40	4.73 ± 1.22	-0.02 ± 0.70	0.9341	13	4.72 ± 1.48	5.04 ± 1.28	0.32 ± 0.58	0.0663	13	5.13 ± 1.81	4.30 ± 1.76	-0.83 ± 1.79	0.1198
BUN/CREA	0.5357	14	71.82 ± 16.92	74.97 ± 18.80	3.16 ± 14.66	0.4345	13	76.16 ± 16.52	81.43 ± 20.90	5.27 ± 18.13	0.315	12	76.33 ± 20.25	75.36 ± 18.06	-0.97 ± 12.17	0.7872
CHE (U/L)	0.3678	14	8378 ± 1767	8086 ± 1629	-293.0 ± 714.9	0.1491	13	8097 ± 1753	8053 ± 1556	-44.15 ± 1079.32	0.8852	13	8402 ± 2058	7632 ± 2910	-770.0 ± 2089.0	0.2086
CK (U/L)	0.0548	14	153.1 ± 111.6	154.4 ± 121.4	1.29 ± 44.34	0.9153	13	113.9 ± 58.2	156.2 ± 111.2	42.23 ± 96.44	0.1403	13	167.2 ± 173.4	140.7 ± 149.3	-26.50 ± 68.55	0.1886
Ca (mmol/L)	0.0601	6	2.47 ± 0.13	2.38 ± 0.24	-0.09 ± 0.13	0.1707	11	2.51 ± 0.11	2.50 ± 0.08	-0.00 ± 0.08	0.8464	10	2.38 ± 0.38	2.50 ± 0.06	0.12 ± 0.37	0.3103
Cl- (mmol/L)	0.2784	6	104.9 ± 3.1	104.3 ± 4.4	-0.57 ± 3.86	0.7339	11	103.7 ± 2.6	105.4 ± 1.6	1.64 ± 3.57	0.1589	10	106.0 ± 2.9	105.5 ± 3.8	-0.49 ± 4.96	0.7618
Crea (µmol/L)	0.4577	14	66.16 ± 11.30	63.99 ± 14.21	-2.16 ± 8.34	0.3491	13	61.73 ± 13.50	63.56 ± 13.58	1.83 ± 14.75	0.6625	13	66.21 ± 12.54	63.34 ± 9.77	-2.87 ± 9.22	0.284
DB (µmol/L)	0.061	14	2.61 ± 1.36	2.11 ± 0.70	-0.50 ± 1.24	0.1544	13	1.88 ± 0.57	2.27 ± 0.59	0.38 ± 0.62	0.044	13	1.94 ± 0.47	1.70 ± 0.65	-0.23 ± 0.73	0.2677
GGT (U/L)	0.4908	14	22.86 ± 20.43	23.07 ± 22.93	0.21 ± 3.42	0.8185	13	15.77 ± 7.85	16.23 ± 8.75	0.46 ± 7.38	0.8253	13	22.77 ± 21.07	22.08 ± 21.83	-0.69 ± 3.95	0.5388
GLOB (g/L)	0.591	14	31.62 ± 4.14	31.02 ± 3.94	-0.60 ± 3.34	0.5135	13	32.93 ± 3.53	31.65 ± 2.56	-1.28 ± 2.36	0.0753	12	32.48 ± 4.20	32.05 ± 2.63	-0.43 ± 2.25	0.5182

GLU (mmol/L)	0.7456	14	4.29 ± 0.50	4.38 ± 0.43	0.09 ± 0.37	0.3985	13	4.20 ± 0.41	4.24 ± 0.58	0.04 ± 0.48	0.7891	12	4.42 ± 0.52	4.37 ± 0.45	-0.06 ± 0.47	0.6848
HDL (mmol/L)	0.3797	14	1.53 ± 0.19	1.53 ± 0.24	0.00 ± 0.17	0.962	13	1.61 ± 0.25	1.53 ± 0.22	-0.08 ± 0.20	0.1922	13	1.52 ± 0.29	1.51 ± 0.23	-0.01 ± 0.16	0.8049
IBIL (μmol/L)	0.8672	14	10.79 ± 6.86	10.71 ± 8.17	-0.09 ± 7.93	0.9684	13	10.32 ± 4.74	9.58 ± 6.62	-0.73 ± 7.03	0.7144	13	8.93 ± 4.75	7.94 ± 4.87	-0.99 ± 5.59	0.5357
K+ (mmol/L)	0.7398	6	4.97 ± 1.02	4.58 ± 0.14	-0.39 ± 0.98	0.3686	11	4.85 ± 0.29	4.42 ± 1.25	-0.43 ± 1.39	0.3316	10	4.89 ± 0.84	4.82 ± 0.41	-0.07 ± 1.13	0.8559
LDL (mmol/L)	0.9419	14	3.50 ± 1.22	3.47 ± 1.16	-0.04 ± 0.22	0.5385	13	3.42 ± 1.28	3.42 ± 1.16	-0.00 ± 0.35	0.9752	12	3.43 ± 1.21	3.36 ± 1.07	-0.07 ± 0.26	0.3631
Mg (mmol/L)	0.3278	6	0.99 ± 0.23	0.91 ± 0.06	-0.08 ± 0.25	0.4818	11	0.94 ± 0.07	0.81 ± 0.27	-0.13 ± 0.30	0.1773	10	0.89 ± 0.08	0.89 ± 0.08	0.00 ± 0.06	0.9212
Na+ (mmol/L)	0.3155	6	132.1 ± 4.4	130.3 ± 8.7	-1.78 ± 4.90	0.4138	11	134.6 ± 1.5	134.6 ± 1.9	0.05 ± 2.40	0.9512	10	134.4 ± 2.0	134.9 ± 1.9	0.56 ± 2.30	0.4619
PHOS (mmol/L)	0.7534	6	1.17 ± 0.10	1.16 ± 0.14	-0.01 ± 0.21	0.9265	11	1.23 ± 0.16	1.11 ± 0.29	-0.12 ± 0.32	0.2614	10	1.19 ± 0.14	1.05 ± 0.34	-0.13 ± 0.42	0.3436
TB (μmol/L)	0.9125	14	13.43 ± 7.36	13.10 ± 8.80	-0.33 ± 7.88	0.8784	13	12.19 ± 4.67	11.86 ± 7.04	-0.33 ± 7.00	0.8676	13	10.88 ± 5.00	9.63 ± 5.21	-1.25 ± 6.11	0.4761
TBA (μmol/L)	0.4401	14	5.62 ± 4.53	5.92 ± 3.68	0.30 ± 3.75	0.7673	13	5.38 ± 2.91	4.69 ± 2.19	-0.69 ± 2.56	0.3534	13	6.46 ± 4.38	5.26 ± 4.06	-1.20 ± 2.95	0.1681
TC (mmol/L)	0.6012	14	5.56 ± 1.32	5.32 ± 1.03	-0.24 ± 0.62	0.1797	13	5.53 ± 1.37	5.39 ± 1.23	-0.14 ± 0.31	0.1353	13	5.49 ± 1.26	5.39 ± 1.13	-0.10 ± 0.32	0.2828
TG (mmol/L)	0.669	14	1.16 ± 0.60	1.13 ± 0.60	-0.03 ± 0.35	0.7727	13	1.11 ± 0.70	0.98 ± 0.49	-0.12 ± 0.39	0.2905	13	1.18 ± 0.68	1.11 ± 0.77	-0.07 ± 0.32	0.4338
TP (g/L)	0.4896	14	76.27 ± 6.72	76.56 ± 3.25	0.29 ± 4.17	0.8018	13	77.72 ± 5.00	76.26 ± 2.62	-1.45 ± 4.56	0.2725	13	76.95 ± 7.02	76.18 ± 3.62	-0.77 ± 4.38	0.5387
UA ((μmol/L)	0.2115	14	251.6 ± 100.1	266.3 ± 93.4	14.74 ± 44.94	0.2414	13	203.1 ± 74.6	243.5 ± 85.9	40.40 ± 88.84	0.127	13	259.0 ± 95.2	244.4 ± 114.9	-14.57 ± 99.02	0.6054
tCO2 (mmol/L)	0.5646	6	26.47 ± 2.49	24.33 ± 2.50	-2.13 ± 4.55	0.3028	11	25.63 ± 0.84	24.42 ± 4.07	-1.21 ± 4.51	0.3944	10	25.44 ± 1.61	25.35 ± 0.89	-0.09 ± 1.16	0.8115
